# Central amygdala circuit dynamics underlying the benzodiazepine anxiolytic effect

**DOI:** 10.1038/s41380-018-0310-3

**Published:** 2018-11-30

**Authors:** Johannes Griessner, Manuel Pasieka, Vincent Böhm, Florian Grössl, Joanna Kaczanowska, Pinelopi Pliota, Dominic Kargl, Barbara Werner, Nadia Kaouane, Sandra Strobelt, Silke Kreitz, Andreas Hess, Wulf Haubensak

**Affiliations:** 1 Research Institute of Molecular Pathology (IMP), Vienna Biocenter (VBC), Campus-Vienna-Biocenter 1, 1030 Vienna, Austria; 2 Bioinformatics and Scientific Computing, Vienna Biocenter (VBC), Dr. Bohr Gasse 3, 1030 Vienna, Austria; 3 Institute of Experimental and Clinical Pharmacology and Toxicology, Friedrich-Alexander University Erlangen-Nuremberg, Fahrstrasse 17, 91054 Erlangen, Germany

**Keywords:** Neuroscience, Psychiatric disorders

## Abstract

Benzodiazepines (BZDs) have been a standard treatment for anxiety disorders for decades, but the neuronal circuit interactions mediating their anxiolytic effect remain largely unknown. Here, we find that systemic BZDs modulate central amygdala (CEA) microcircuit activity to gate amygdala output. Combining connectome data with immediate early gene (IEG) activation maps, we identified the CEA as a primary site for diazepam (DZP) anxiolytic action. Deep brain calcium imaging revealed that brain-wide DZP interactions shifted neuronal activity in CEA microcircuits. Chemogenetic silencing showed that PKCδ^+^/SST^−^ neurons in the lateral CEA (CEAl) are necessary and sufficient to induce the DZP anxiolytic effect. We propose that BZDs block the relay of aversive signals through the CEA, in part by local binding to CEAl SST^+^/PKCδ^−^ neurons and reshaping intra-CEA circuit dynamics. This work delineates a strategy to identify biomedically relevant circuit interactions of clinical drugs and highlights the critical role for CEA circuitry in the pathophysiology of anxiety.

## Introduction

Anxiety disorders comprise a major unmet medical problem affecting a large population in western industrialized nations [1–3], which creates a significant socioeconomic cost [4, 5]. While anxiolytic drugs can relieve symptoms, they can also cause considerable side effects. Benzodiazepines (BZDs) have been in clinical use for decades and exhibit not only anxiolytic, but also amnestic, anti-convulsant, sedative, muscle relaxant, and addictive properties [6–8]. BZDs act as positive allosteric modulators on compatible GABA_A_ receptors [9], but little is known about which neuronal circuits mediate their anxiolytic effects. Such knowledge could reveal critical target circuits for novel, directed therapeutic interventions and contribute to our understanding of the pathophysiology of anxiety. Here, we combined publicly available connectome data from the Allen Mouse Brain Atlas [10] (http://connectivity.brain-map.org) with a limbic-system-wide immediate early gene (IEG) screen to map the effects of diazepam (DZP) on anxiety-related brain activity. Subsequent ex vivo electrophysiology, opto- and chemogenetic manipulations, in vivo calcium imaging, blood-oxygen-level dependent (BOLD) fMRI imaging, and cell type specific transcriptome profiling suggest inhibitory gating by central amygdala (CEA) interneurons as a critical mechanism for the anxiolytic effects of BZDs.

## Methods and materials

### Subjects

Male C57BL6/J mice (Charles River) were used for the c-Fos network analysis and in the fMRI experiment. PKCδ::GluClα-CRE BAC (PKCδ::Cre, mmrrc #11559) transgenic mice were used for DREADD experiments. SST-IRES-Cre mice (SST::Cre, Jackson #013044) and PKCδ::Cre mice were crossed to Rosa::loxP-STOP-loxP-td-Tomato (Jackson #007905) mice. The resulting offspring were then used for neural population sequencing and electrophysiology. SST-IRES-Cre mice, PKCδ::Cre mice and C57BL6/J mice (all males) were used for deep brain calcium imaging experiments. Mice were group housed at 21 °C in a 14 h light and 10 h dark cycle (day starting at 6:00 a.m.), and all tests were performed during the light period. Food and water were provided ad libitum. All animal experiments were performed in accordance with institutional guidelines and were approved by the respective Austrian (BGBl nr. 501/1988, idF BGBl I no. 162/2005) and European (Directive 86/609/EEC of 24 November 1986, European Community) authorities and covered by the license MA58/002220/2011/9.

### Behavioral tests

A standard elevated plus maze (EPM) assay was used, in which animals were placed in the center zone facing an open arm and allowed to freely explore the apparatus for 5 min. Intraperitoneal (i.p.) injections were administered 30 min before each session. For the c-Fos screen, 15 min after injection animals were placed in a novel chamber for 10 min where only the appropriate cohorts received 10 foot-shocks, 0.5 mA, of 1 s at randomized intervals of 20–100 s. They were then transferred back to their home cage where they either stayed for 5 min before they were exposed to the EPM or for 95 min until euthanasia. All sessions were recorded and analyzed using ANY-maze (Stoelting Europe). For calcium imaging, mice were fear-conditioned to sound (3 kHz) and context (5 tone-foot shock pairings presented over 10.5 min). After 1 week, they were re-exposed to the conditioning context on two separate days to measure anxiety. Before each session, they received i.p. injections of either DZP (1 mg/kg, day 1) or saline (day 3). Behavior was analyzed using Matlab programs (R2015b; MathWorks, Natick, MA, USA) and Ethovision XT 8 (Noldus Information Technology, Wageningen, the Netherlands).

### c-Fos network analysis

**Fig. 1:**
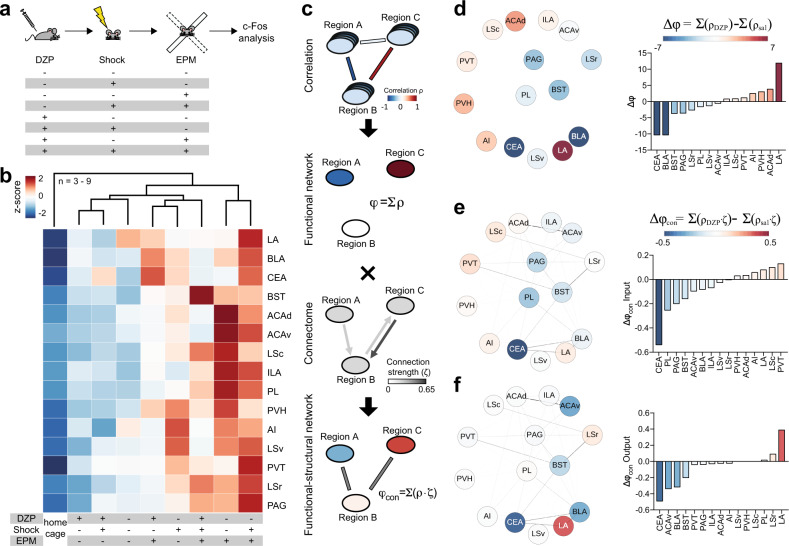
Mapping the interaction of DZP and anxiety in the limbic system. **a** After i.p. injection of saline or DZP, mice were placed back into their home cage or exposed to a series of foot shocks, a 5 min EPM session or both. Brains were fixed 2 h after injection. **b** Hierarchical clustering of region-wise *z*-scored c-Fos expression for 15 limbic regions in all behavioral states. **c** Schematic of analysis workflow. Pairwise functional correlations between regions were calculated for DZP and saline groups over all anxiety conditions. Summation of all these correlations (*φ*) shows how strongly any region correlates with the network. Next, *φ* was scaled to the first-order anatomical connections between regions using the Allen Mouse Brain Connectome (AMBC). The resulting measure (*φ*_con_) indicates how well any region correlates with its neighbors. **d**–**f** Effects of DZP on *φ* and *φ*_con_. Left: network representation of all regions analyzed. Nodes are color-coded for effect size. Edges represent undirected anatomical connections taken from AMBC. Right: rank-ordered effect size for each region. **d** Δ*φ* shows the difference of *φ* between DZP and saline conditions, indicating change in functional network-correlation by DZP treatment for each region. The CEA, BLA, and LA showed the greatest Δ*φ* in absolute terms. **e, f** For Δ*φ*_con_, Δ*φ* was scaled for both incoming (**e**) and outgoing (**f**) connections. The CEA showed the greatest Δ*φ*_con_ in both measures, indicating a strong decoupling from its neighbors. ACAd anterior cingulate area, dorsal part; ACAv anterior cingulate area, ventral part; AI agranular insular area, BLA basolateral amygdalar nucleus, BST bed nuclei of the stria terminalis, CEA central amygdalar nucleus, ILA infralimbic area, LA lateral amygdalar nucleus, LSc lateral septal nucleus, caudodorsal part; LSr lateral septal nucleus, rostroventral part; LSv lateral septal nucleus, ventral part; PAG periaqueductal gray, PL prelimbic area, PVH paraventricular hypothalamic nucleus, PVT paraventricular nucleus of the thalamus

First, c-Fos expression values were averaged within each treatment group to yield one value for each group and region. For each region, *z*-scores over all treatment groups were calculated. The home-cage group was only included in the hierarchical clustering in Fig. 1b, but not for any subsequent analysis. Second, the *z*-scores were used to compute correlation matrices between all regions in without-drug (“saline”, “saline & EPM”, “saline & shock”, “saline & shock & EPM”) and with-drug (“DZP”, “DZP & EPM”, “DZP & shock”, “DZP & shock & EPM”) conditions. A four-dimensional vector was built for each region in both conditions and their Pearson correlation (*ρ*) calculated. Third, the sum of all pairwise correlations with each region was calculated (*φ* = Σ*ρ*), and the effect of the drug (DZP) analyzed using the difference of *j* between drug and no drug measures (i.e. Δ*φ* = *φ*_DZP_ – *φ*_saline_). Fourth, the Allen Mouse Brain Connectome (AMBC) was used to weight correlations according to anatomical connection strength. A subset of the AMBC was used that contained the regions for which we had collected c-Fos expression data. Next, this connectome subset was normalized to the outgoing edges of each region (Σζ_out_ = 1, ζ_out_ being the connection strength of outgoing connections). For each region, the sum of all scaled correlations to all its first-order neighbors was calculated (*φ*_con_ = Σ*ρζ*), conveying both functional and structural information. The effect of DZP was again measured by taking the difference between both states (i.e. Δ*φ*_con_ = *φ*_con(DZP)_–*φ*_con(saline)_). Because the connectome contains directional information, the node analysis can be performed based either on outgoing edges (i.e. *φ*_con_ Output) or incoming edges (i.e. *φ*_con_ Input).

### Neuronal population sequencing

Offspring (males, 2–5 months old) from crosses of Rosa::loxP-STOP-loxP-td-Tomato to PKCδ::Cre or SST::Cre were decapitated and the CEA extracted in ice-cold Hibernate A Low Fluorescence solution (BrainBits) from the brain and enzymatically dissociated (Papain Dissociation System, Worthington Biochem). FACS sorting retrieved approximately 10^3^ td-Tomato^+^ cells. SMARTer® smRNA-Seq Kit for Illumina® (Clontech, 78100 Saint-Germain-en-Laye, France) was used to prepare libraries. Deep sequencing was performed on a HiSeq 2500 system (Illumina, San Diego, USA), followed by standard bioinformatical analysis.

### Ex vivo electrophysiology

SST::tdTomato mice (male, 2–3 months) were euthanized and 300-µm-thick brain slices cut in dissection buffer and subsequently transferred to oxygenated aCSF. Recordings from identified SST^+^ or SST^−^ neurons were performed in whole-cell voltage-clamp configuration (−70 mV). After a 5 min baseline, DZP (10 μM) was added to the bath. Two-minute bins of sIPSCs from before and after addition of DZP were analyzed for each cell.

### Deep brain calcium imaging

A total of three animals (CEAl SST^+^ /PKCδ^−^), four animals (CEAl PKCδ^+^/SST^−^) and four animals (CEm) were imaged using a nVista HD 2.0 in vivo Rodent Brain Imaging System (Inscopix, Palo Alto, USA), with GLP-0561 microendoscopic fibers implants. Behavioral control, Ca^2+^, and behavioral videos were recorded on a fully synchronized custom built setup, running on Anymaze (Stoelting, Wood Dale, USA), Arduino 2.0 scripts, and nVistaHD v2.0.32 software, respectively. Behavioral data were analyzed in Anymaze, calcium imaging data were acquired at 20 fps, motion compensated and with MOSAIC v1.2 software (Inscopix, Palo Alto, USA) and custom ImageJ scripts. Units were extracted by session-wise Δ*F*/*F*_0_ normalized recordings by PCA/ICA (MOSAIC v1.2) performed across all recording sessions. These automatically identified units were visually curated for quality and stability, yielding stably registered and identified units across both recording sessions. Calcium events were detected from 2 Hz low pass filtered traces (Fig. 3b, right, bottom) at a threshold >6 SD and decay time *τ* > 0.5 s (Fig. 3b, right, top). Units were projected onto mean movies (Fig. 3b, left). Neuronal activity was computed as the cumulative amplitude of above threshold calcium events (event score) for each neuron in each session (Fig. 3c, top). This workflow yielded a total 24 units from two (CEAl SST^+^/PKCδ^−^), 14 units from three animals (CEAl PKCδ^+^/SST^−^), and 33 units from four animals (CEm) with significant activity. Population activity represents the cell-wise event scores for each session. Units were classified as increasing (Δ_event score BZD-saline_ > 0), decreasing (Δ_event score BZD-saline_ < 0), or unaffected (Δ_event score BZD-saline_ = 0), respectively (Fig. 3c, bottom).

### Statistical analysis

All statistical tests are indicated in the figures legends. The tests were two-tailed and corrected for multiple comparisons whenever applicable. Whenever data were found to be not normally distributed, non-parametric tests were used. Otherwise normality was assumed. When the significance is not noted, the test did not reach statistical significance. All statistical analyses were performed in Graph Pad Prism ® (Version 7) unless indicated otherwise.

## Results

### Interaction of BZDs and anxiety in limbic circuits

To screen for the interaction of BZDs with brain anxiety states, we administered DZP at an anxiolytic but not sedative dose (1 mg/kg; Supplementary Fig. S1) and mapped limbic c-Fos expression after exposure to different anxiogenic stimuli, ranging from a 5 min long EPM session (a BZD sensitive, mildly anxiogenic environment by itself [11–13] and used for functional analysis at a later stage) to a series of ten 0.5 mA foot shocks (which promote anxiety [14–18]), or a combination of both (Fig. 1a) [19]. We reasoned that studying BZDs in the challenged brain most closely mimics situations that require therapeutic intervention. Applying a range of anxiogenic stimuli (low-anxiety EPM versus high-anxiety inescapable foot shocks) should maximize the contribution of anxiety over other behavioral states to co-vary with brain activity. However, using different anxiogenic exposures (acrophobia versus inescapable, imminent danger, and their combination) minimizes the contribution of purely sensory components of any single anxiogenic experience. This should help dissociate anxiety-related BZD effects on brain activity from non-anxiety side effects in later analyses.

Mice were euthanized 2 h after drug injection (i.e. 90–95 min after the last anxiogenic stimulus). After preparation, the brains were analyzed for c-Fos expression as a proxy for neuronal activity induced by shock, EPM, and/or drug treatment. We quantified c-Fos levels from 15 limbic brain regions by semi-automated immunohistochemistry (IHC). Next, *z*-scores of c-Fos expression were calculated for each region to capture treatment effects. Hierarchical clustering of these data (Fig. 1b) indicated an interaction of DZP with each anxiety state. All DZP groups clustered in the same branch, and no DZP group clustered in the same two-leafed branch as its corresponding saline group. Indeed, statistical analyses revealed an overall drug effect for each condition (Supplementary Fig. S2; DZP mostly decreased c-Fos levels, reflecting its central nervous system inhibitory properties).

Brain regions do not operate in isolation, rather they are highly interconnected state-specific functional networks. In our case, such functional interactions can be used to locate sites with particularly strong interactions between drugs and anxiety. So, we emulated and compared anxiety-related functional networks in saline and drug states derived from c-Fos brain activity patterns (Fig. 1b). In this analysis, we modeled the coupling of each region by correlating its activity with every other brain region in either the drug or saline conditions across all four anxiety states (Fig. 1c; Supplementary Fig. S3a, b). The sum of all these correlations (*φ*) gives a measure of how well activity in a given region correlates with the rest of the network, while the difference (Δ*φ*) between drug and saline conditions shows how strongly DZP treatment alters this correlation (Fig. 1d; Supplementary Fig. S3c). The three amygdala nuclei (the lateral, basolateral, and CEA) showed the greatest change in correlation in absolute terms, suggesting that the net global interaction of DZP with anxiety in this network is strongest at these sites.

We next sought to extract those sites that could be direct drug targets. Direct drug effects should perturb local physiological connectivity and decouple these sites from their direct anatomical neighbors. We therefore fused our c-Fos data with structural connectivity from the Allen Mouse Brain Atlas [10]. We weighted the between-region correlations obtained before by multiplying each correlation coefficient with its corresponding connectivity score from the Allen Mouse Brain Atlas for both incoming and outgoing connections (Fig. 1c, e, f; Supplementary Fig. S3d–f). In analogy to *φ*, the sum of all these weighted correlations per region (*φ*_con_) measures the correlation of activity in any region with its anatomical neighbors, while the difference Δ*φ*_con_ between the DZP and saline states indicates how strongly the drug changes this local correlation. Within the investigated network, a high Δ*φ*_con_ in absolute terms was found for the amygdala and several other cortical and subcortical structures, reflecting a DZP-induced decoupling of these regions. Interestingly, the CEA showed the greatest drug-induced decorrelation for both incoming and outgoing connections (Fig. 1e, f; Supplementary Fig. S3g), identifying it as a potential target of BZD anxiolytic activity.

### Modulation of CEA microcircuitry by BZDs

**Fig. 2:**
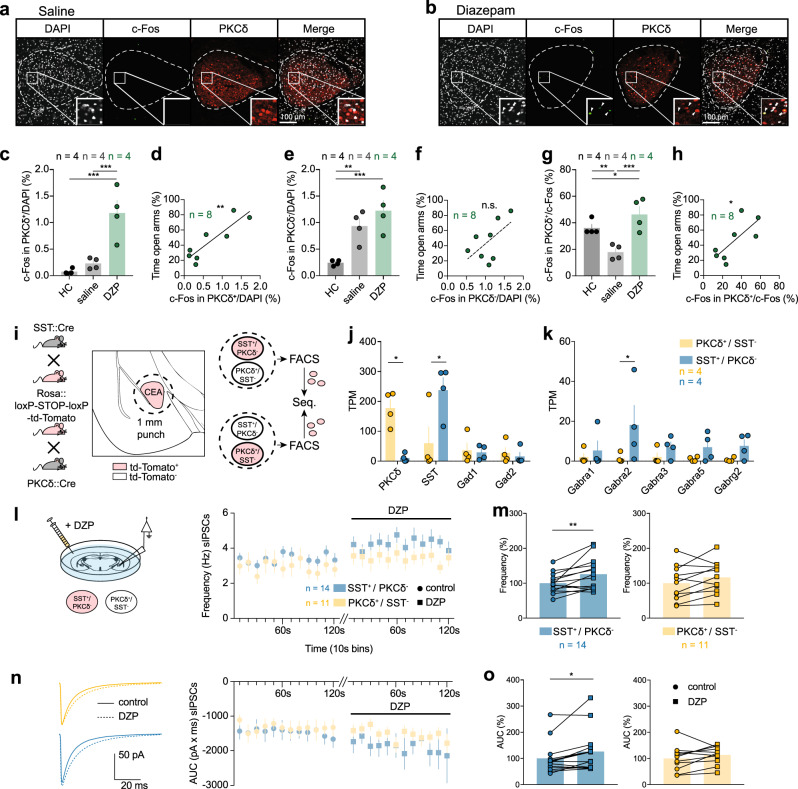
DZP disinhibits CEAl PKCδ^+^ neurons. **a**, **b** Confocal scans with colocalizing c-Fos and PKCδ IHC in CEAl after DZP treatment and EPM exposure. Arrowheads indicate c-Fos^+^ cells. **c–h** Compared to saline controls, DZP increases c-Fos expression in PKCδ^+^ (**b** one-way ANOVA F [2, 9] = 18.11, ***P*** = 0.0007; two-stage linear step-up procedure of Benjamini, Krieger, and Yekutieli) but not PKCδ^−^ cells (**e** one-way ANOVA F [2, 9] = 12.86, *P* = 0.0023; two-stage linear step-up procedure of Benjamini, Krieger, and Yekutieli) and shifts the ratio of c-Fos expression towards PKCδ^+^ cells (**g** one-way ANOVA F [2, 9] = 11.92, *P* = 0.003; two-stage linear step-up procedure of Benjamini, Krieger, and Yekutieli). Note that while PKCδ^−^ neurons are activated by saline injection and EPM exposure alone, PKCδ^+^ neurons are silent without DZP treatment. The expression of c-Fos in PKCδ^+^ (**d** Pearson *r*, *R*^2^ = 0.7777, *P* = 0.0038; **h** Pearson *r*, *R*^2^ = 0.546, *P* = 0.0362) but not PKCδ^−^ cells (**f** Pearson *r*, *R*^2^ = 0.3355, *P* = 0.1324) correlates with EPM open arm time. **i** CEA PKCδ^+^/SST^−^ and SST^+^/PKCδ^−^ cells were isolated by microdissection and FACS from PKCδ::Cre or SST::Cre crosses to Cre-dependent Rosa::td-Tomato reporter lines and submitted to mRNA sequencing. **j** Expression levels (transcripts per million reads mapped, TPM). Marker genes for cell type but not for GABA signaling show differential expression in the FACS sorted populations (RM two-way ANOVA F_interaction_ [3, 18] = 14.1, *P* < 0.0001; F_gene_ [3, 18] = 9.889, *P* = 0.0004; F_cell type_ [1, 6] = 0.007385, *P* = 0.9343; two-stage linear step-up procedure of Benjamini, Krieger, and Yekutieli). **k** Expression levels (transcripts per million reads mapped, TPM) of canonical BZD binding GABA_A_-receptor subunits. The α_2_ subunit of the GABA_A_ receptor is more strongly expressed in SST^+^/PKCδ^−^ cells (RM two-way ANOVA F_interaction_ [4, 24] = 0.8751, *P* = 0.4934; F_gene_ [4, 24] = 0.8021, *P* = 0.5358; F_cell type_ [1, 6] = 9.957, *P* = 0.0197); two-stage linear step-up procedure of Benjamini, Krieger, and Yekutieli). **l**–**o** Patch-clamp recordings from CEAl SST^+^/PKCδ^−^ and PKCδ ^+^/SST^−^ neurons from a total of eight animals. **l** Left: Schematic of experiment. Right: Binned [10 s] frequency of sIPSCs of both CEAl populations before and during DZP application. **m** Frequency of sIPSCs normalized to group average at baseline. Left: DZP increased the frequency of sIPSCs in SST^+^/PKCδ^−^ neurons (Wilcoxon matched-pairs signed rank test: sum of signed ranks = 89, number of pairs = 14, *P* = 0.0031). Right: No change was detected in PKCδ^+^/SST^−^ neurons (Wilcoxon matched-pairs signed rank test: sum of signed ranks = 36, number of pairs = 11, *P* = 0.1230). **n** Left: Average traces of IPSCs of both CEAl populations. Right: Binned [10 s] AUC of sIPSCs of both CEAl populations before and during DZP application. **o** AUC of sIPSCs normalized to group average at baseline. Left: DZP potentiates sIPSCs on SST^+^/PKCδ^−^ neurons (Wilcoxon matched-pairs signed rank test: sum of signed ranks = 71, number of pairs = 14, *P* = 0.0245). Right: No change was detected in PKCδ^+^/ SST^−^ neurons (Wilcoxon matched-pairs signed rank test: sum of signed ranks = 34, number of pairs = 11, *P* = 0.1475). Bars are means ± s.e.m. Significance levels between groups at *P/*Q* < 0.05, **P/*Q* < 0.01, ***P/*Q* < 0.001. For *m* and *o* significance levels between groups were at *P < 0.025. In **d**, **f**, and **h** lines are linear regression lines. GABA_A_ receptor subunits: Gabra1-3,5 – α_1-3,5_, Gabrg2 – γ_2_

The CEA is an essential hub for fear and anxiety processing and its circuit architecture and function have been described in great detail [20–22]. Its lateral subdivision (CEAl) controls behavioral responses to aversive stimuli by inhibitory gating of the medial CEA (CEAm) output to the brainstem [21], a process mediated largely by CEAl PKCδ^+^ neurons [22]. We thus investigated the effect of systemic BZDs on inhibitory gating in the CEA. Using double-label IHC for c-Fos and PKCδ on EPM-challenged mice (Fig. 2a, b), we found selectively increased activity measured by c-Fos in PKCδ^+^ but not PKCδ^−^ cells (Fig. 2c, e, g). Further, activity in PKCδ^+^ cells but not PKCδ^−^ cells was positively correlated with open arm time in the EPM (Fig. 2d, f, h). Given the inhibitory action of BZDs, this increase in PKCδ^+^ activity might seem paradoxical. However, CEAl PKCδ^+^ neurons are under inhibitory control of CEAl SST^+^ (largely identical to PKCδ^-^) [23] neurons, the other major class of antagonistically wired GABA-ergic neuronal populations (Supplementary Fig. S9). We concluded that differential BZD sensitivity of these antagonistically wired populations might shift CEA GABA-ergic tone that ultimately disinhibits PKCδ^+^/SST^−^ neurons (Fig. 2g).

Next, we investigated if BZDs could modulate neuronal processing directly in the CEA. Both α and γ GABA_A_-receptor subunits, which only jointly allow BZD binding [9], are present in the CEA [24] and BZD infusions into this nucleus can be anxiolytic [25, 26]. To evaluate the GABA_A_ subunit distribution in CEAl, we FACS-sorted the two major CEAl populations from Cre-dependent tdTomato reporter lines crossed to either SST::Cre or PKCδ::Cre (Fig. 2i–k; Supplementary Fig. S4). Transcriptome profiling indicated that those GABA_A_-receptor subunits that are highly involved in BZD-binding [9] were expressed in SST^+^/PKCδ^−^ neurons at higher levels (Fig. 2k), while this was not the case for the remaining GABA_A_-receptor subunits (Supplementary Fig. S4). Notably, a GABA_A_-receptor subunit implicated in anxiolytic BZD action (GABA_A_-α_2_) [27] was expressed predominantly by SST^+^/PKCδ^−^ neurons (Fig. 2k). We also found significant asymmetry in the expression of the GABA_A_-γ_1_ subunit (Supplementary Fig. S4), but BZDs exhibit only reduced activity on receptors containing γ_1_ or γ_3_ subunits [9, 28, 29]. In line with this asymmetric receptor-subunit expression, recordings from CEAl neurons ex vivo showed that bath application of DZP increased both frequency and size of spontaneous inhibitory postsynaptic currents (sIPSCs) of SST^+^/PKCδ^−^ neurons, an effect that could not be detected in PKCδ^+^/SST^−^ neurons under these conditions (Fig. 2l–o). The increase in frequency of sIPSCs (Fig. 2l, m) might reflect increased presynaptic inhibition by PKCδ^+^/SST^−^ neurons, while the increase in size (Fig. 2n, o) is in line with a direct postsynaptic effect of DZP on GABA_A_ receptors.

Thus, sensitivity of SST^+^/PKCδ^−^ neurons to DZP provides a simple mechanistic explanation for BZD-induced disinhibition of PKCδ^+^/SST^−^ neurons, reflected by their increased c-Fos expression (Fig. 2a–h) and resulting in increased sIPSC-frequency in SST^+^/PKCδ^−^ neurons (Fig. 2l, m). However, these findings do not exclude alternative scenarios involving additional neuronal populations in CEAl, and the current electrophysiological data do not rule out BZD effects on PKCδ^+^/SST^−^ neurons. We propose that these effects on local GABA-ergic signaling synergize with global BZD network effects that ultimately shift CEA circuitry dynamics (Supplementary Fig. S9) to facilitate inhibitory gating of CEA fear output.

**Fig. 3:**
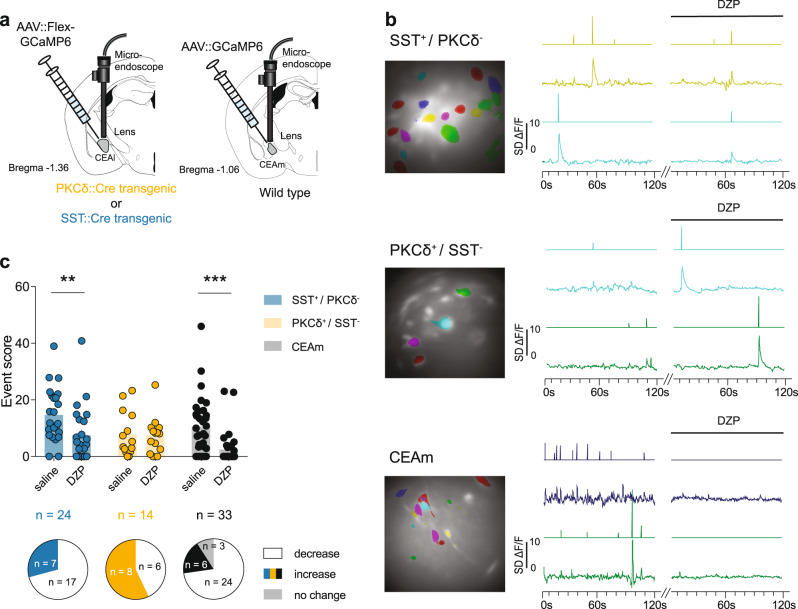
DZP modulates CEA circuit dynamics in vivo. **a** Targeted imaging of central amygdala neuronal populations. After AAV-mediated Cre-dependent expression of GCaMP6, SST^+^/PKCδ^−^, and PKCδ^+^/SST^−^ neurons were imaged in CEAl. CEAm neurons were imaged after AAV-mediated expression of GCaMP6. **b**, **c** Calcium imaging of CEA neurons after saline or DZP treatment. Mice were placed in a conditioned aversive context after injection of saline and DZP on two separate days and neuronal activity was recorded. **b** Modulation of individual CEA neurons by DZP. Left: Exemplary mean images and registered units for each cell type. Right: Raw calcium traces (bottom) and events (top) from two exemplary units from each recording. Color code of the traces (right) corresponds to color of the units (left). **c** Modulation of population activity by DZP. Top: Event score of SST^+^/PKCδ^−^ units (24 neurons extracted from *n* = 3 animals imaged), and PKCδ^+^/SST^−^ units (14 neurons extracted from *n* = 4 animals) and units in CEAm (33 neurons extracted from *n* = 4 animals). DZP suppressed activity in CEAl SST^+^/PKCδ^−^ and CEAm, but not in CEAl PKCδ^+^/SST^−^ neurons (RM two-way ANOVA F_interaction_ [2, 68] = 1.885, *P* = 0.1597; F_drug_ [1, 68] = 10.33, *P* = 0.002; F_cell type_ [2, 68] = 4.777, *P* = 0.0115; Two-stage linear step-up procedure of Benjamini, Krieger, and Yekutieli). Bottom: Proportion of neurons with increased, decreased, or unchanged activity under DZP treatment was linked to cell type (*χ*^2^ = 6.221, df = 2, *P* = 0.0446). Bars are means ± s.e.m. Significance levels between groups at *Q  <  0.05, **Q < 0.01, ***Q < 0.001

To investigate such BZD-driven reshaping of CEA circuit dynamics in vivo, we performed calcium imaging in freely behaving animals expressing GCaMP6 in either CEAl SST^+^/PKCδ^−^, PKCδ^+^/SST^−^, or CEAm neurons (Fig. 3a). Mice were fear conditioned and received DZP or saline injections before being placed in the conditioning context on two separate days, which served as an anxiogenic environment. We monitored calcium traces of neurons registered over both sessions (Fig. 3b, bottom traces). Neural activity was measured as an event score representing the cumulative amplitude of all calcium spikes (Fig. 3b, top traces) for each neuron. While SST^+^/PKCδ^−^ population activity was reduced by DZP, population activity of PKCδ^+^/SST^−^ was unaffected, reflecting a larger effect of DZP on the SST^+^/PKCδ^−^ population (Fig. 3c top). Further, CEAm population activity was strongly reduced by DZP (Fig. 3c top). While a majority of SST^+^/PKCδ^−^ and CEAm neurons largely showed suppressed activity after DZP application, the majority of PKCδ^+^/SST^−^ exhibited increased activity (Fig. 3c, bottom). Overall, these data suggest that BZDs shift the balance of activity in CEA from SST^+^/PKCδ^−^ and CEAm neurons to PKCδ^+^/SST^−^ cells, which results in a net-suppression in amygdala fear output (Supplementary Fig. S9).

### CEA circuits are essential for the BZD anxiolytic effect

**Fig. 4:**
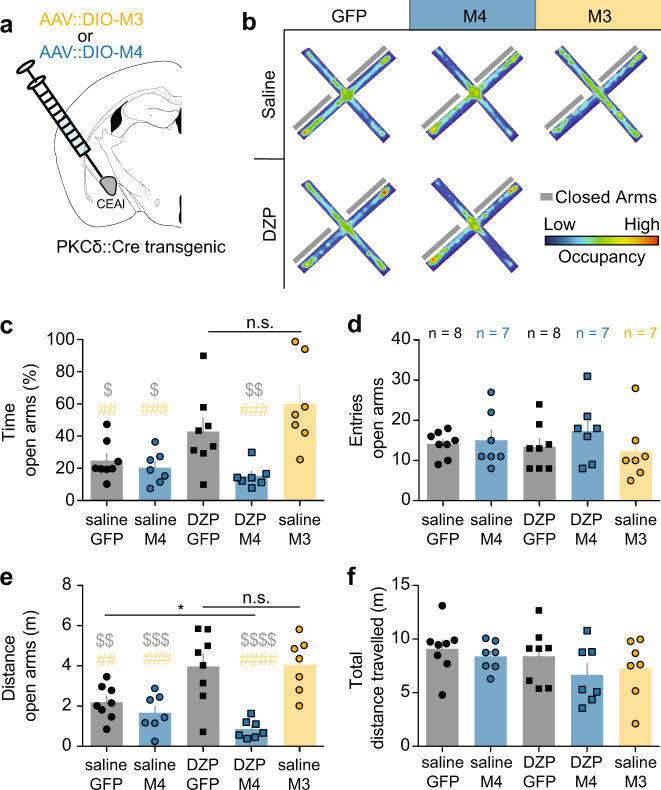
CEAl PKCδ^+^ cell activity is necessary and sufficient for the anxiolytic effect of DZP. **a** AAV-mediated Cre-dependent expression of DREADDs AAV::DIO-M4 (M4) or AAV::DIO-M3 (M3) in CEAl neurons of PKCδ::Cre transgenic mice. **b–f** Interaction of DZP and CEAl PKCδ^+^/SST^−^ neuron activity in the EPM. All mice received i.p. CNO (5 mg/kg) in addition to either DZP (1 mg/kg) or saline. **b** Average occupancy plots for each experimental condition. **c** DZP increased the time spent in the open arms. Inhibiting CEAl PKCδ ^+^/SST^−^ neurons did not significantly alter open arm time by itself but reverted the anxiolytic effect of DZP. Activation of CEAl PKCδ ^+^/SST^−^ neurons seemed to mimic the effect of DZP (one-way ANOVA F [4, 32] = 7.603, *P* = 0.0002; two-stage linear step-up procedure of Benjamini, Krieger, and Yekutieli). **d** Entries to the open arms were unchanged in all groups (one-way ANOVA F[4, 32] = 0.6223, *P* = 0.6499; two-stage linear step-up procedure of Benjamini, Krieger, and Yekutieli). **e** The distance travelled in the open arms showed a pattern similar to the time in the open arms (one-way ANOVA F [4, 32] = 10.57, *P* < 0.0001; two-stage linear step-up procedure of Benjamini, Krieger, and Yekutieli). **f** The total distance travelled was unchanged in all groups (one-way ANOVA F [4, 32] = 1.194, *P* = 0.3325; two-stage linear step-up procedure of Benjamini, Krieger, and Yekutieli). Bars are means ± s.e.m. Significance levels between groups (*) to DZP GFP ($) and saline M3 (#) groups at */$/# *Q* < 0.05, **/$$/##*Q* < 0.01, ***/$$$/### *Q* < 0.001, ****/$$$$/#### *Q* < 0.0001

Since our data positively associate CEl PKCδ^+^/SST^−^ neurons with BZD treatment, we investigated a potential direct functional role CEl PKCδ^+^/SST^−^ activity in the BZD anxiolytic effect. We injected AAV_2/5_-hSyn-DIO-hM4D-mCherry-WPRE-hGh (AAV::DIO-M4, virus expression shown in Supplementary Fig. S7) bilaterally into the CEAl of PKCδ::CRE mice, which allows for specific, functional silencing of PKCδ^+^/SST^−^ neurons following systemic administration of clozapine-n-oxide (CNO, 5 mg/kg, an effective dose that does not impair motor behavior [30, 31] (Fig. 4a). Control animals, PKCδ::CRE mice injected into the CEAl with AAV_2/5_-EF1α-DIO-GFP-WPRE-hGh (AAV::DIO-GFP; Supplementary Fig. S7) and which also received CNO, responded to DZP application (1 mg/kg) by spending more time in the open arms of the EPM (Fig. 4b, c). This effect reverted to baseline levels by simultaneous silencing of PKCδ^+^/SST^−^ neurons (Fig. 4b, c), indicating that PKCδ^+^/SST^−^ activity is required for the DZP anxiolytic effect. In order to test whether the converse was true, we used a similar approach and specifically activated PKCδ^+^/SST^−^ neurons in PKCδ::CRE mice injected with AAV_2/5_-hSyn-DIO-hM3D-mCherry-WPRE-hGh (AAV::DIO-M3; Supplementary Fig. S7) into the CEAl. Following CNO administration, these mice showed greatly increased open arm time, phenocopying the behavioral effect of DZP administration (Fig. 4b, c) and replicating the anxiolytic effect of optogenetic PKCδ^+^/SST^−^ activation [32]. Distance travelled in the open arms reflected open arm time (Fig. 4e), while open arm entries and total mobility remained unchanged in all treatment groups (Fig. 4d, f). These data indicate that both chemogenetic manipulation and DZP selectively affected anxiety behavior in this assay.

### BZDs interact with the relay of aversive signals through the CEA

While the DREADD experiments demonstrated that CEAl microcircuitry is critical for the anxiolytic effect of DZP, we wanted to assess whether DZP could reverse an anxiogenic brain state produced by functionally relevant macrocircuitry involving the CEAl. As a central hub for fear and anxiety, the CEA integrates aversive signals from various cortical and subcortical inputs and controls aversive states through its widespread forebrain and brainstem targets [33]. We predicted that BZDs may reduce anxiety by interfering with the relay of these aversive signals in this network.

To test this hypothesis, we optogenetically stimulated aversive signals from the paraventricular nucleus of the thalamus (PVT), an anxiogenic input to CEAl [19, 34], and monitored the provoked modulations of global brain interactions by BOLD fMRI. We injected male C57BL/6J mice with AAV_2/5_-hSyn-hChR2(H134R)-eYFP-WPRE-hGh or AAV_2/5_-hSyn-eGFP-WPRE-hGh (AAV::ChR2, AAV::GFP; virus expression shown in Supplementary Fig. S8; in these viruses, the synapsin promoter conveyed neuron-specific expression of ChR2 and GFP, respectively) into the PVT and implanted optical fibers above the right CEAl to confer projection-specific activation (Supplementary Fig. S5a, fiber location shown in Supplementary Fig. S8). Graph-theoretical analyses showed that optogenetic stimulation lead to specific modulations in functional brain networks under DZP treatment (Supplementary Fig. S6b, color coded brain regions) and particularly in statistically significant differences in functional connectivity within limbic functional subnetworks (Supplementary Fig. S6e). Overall, the functional network induced optogenetically by PVT-CEAl activation reverted after DZP administration (Supplementary Fig. S6b, compare ChR2 DZP with GFP saline network and clustering similarity Supplementary Fig. S6c as well as the high overall network similarity Supplementary Fig. S6d). DZP administration rearranged intraamygdala connectivity (especially the bilateral connectivity of the amygdala) and increased amygdala–brainstem (PAG) interactions in the GFP, as well as in the ChR2 group, while PVT-to-CEAl optogenetic activation alone (ChR2) induced the converse, partially antagonizing the BZD effect (Supplementary Fig. S6c, e). In summary, we conclude that BZDs antagonize the relay of optogenetically induced aversive signals through the CEA by facilitating inhibitory gating.

## Discussion

In summary, we investigated the neuronal mechanism underlying the anxiolytic effect of DZP as a prototypical BZD. We combined publicly available information from the AMBC with a functional network analysis based on anxiety-related c-Fos data. While c-Fos screens are not typically analyzed in the context of structural network information, our results suggest that such an integrated analysis may facilitate the identification of drug target sites. This strategy suggested several key regions for the anxiolytic BZD effect, including some which have previously been implicated in it, e.g. the CEA [25, 26] (Supplementary Fig. S9) and BLA (Supplementary Fig. S10). While these regions all might contribute to BZD-induced anxiolysis, we followed up on the CEA as it ranked highest in our screen (measured by Δ*φ*_con_) in this study.

Subsequent ex vivo electrophysiological recordings, in vivo calcium imaging and chemogenetic manipulations in freely behaving mice suggested that shifting CEA circuit dynamics toward PKCδ^+^/SST^−^ neurons promotes the BZD anxiolytic effect. This may be locally facilitated by an asymmetric expression of BZD-sensitive GABA_A_ receptors that shifts activity to PKCδ^+^/SST^−^ neurons, while suppressing SST^+^/PKCδ− cells, which have been associated with aversive states (Supplementary Fig. S9) [23, 35]. These data could resolve the previous paradoxical observation that BZDs increase neuronal activity in the CEA [36] and provide a mechanism for the behavioral effect of BZDs observed after local CEA infusions [25, 26]. Projection-specific optogenetic fMRI and pharmacology allowed embedding these findings in a mesoscale circuit context, in line with these local BZD effects antagonizing the relay of aversive signals through the CEA (Supplementary Fig. S9). Indeed, this scenario is supported by in vivo calcium imaging data showing that BZDs strongly suppress CEAm activity (Fig. 3c).

The current data, while in agreement with previous data on the CEAl in its entirety [21] and on the anxiolytic activity of CEAl PKCδ^+^/SST^−^ neurons [32], stand in contrast to recent findings [37], which positively correlate CEAl PKCδ^+^/SST^−^ activity with conditioned fear states. This discrepancy suggests a complex scenario in which CEA circuity differentially processes phasic fear stimuli vs. tonic anxiety states. Given all data at hand, we believe the most likely interpretation concludes that BZDs affect anxiety both by binding to CEA neurons directly, as well as through indirect global network effects that modulate the tone of CEA circuit components to ultimately counteract anxiety states in CEA [19] and dampen amygdala output. The circuit architecture suggests [20] (Supplementary Fig. S9) that this could be mediated by inhibitory gating of CEAm output by the CEAl [21], potentially driven by PKCδ^+^/SST^−^ activity [22, 32].

To evaluate the translational potential of our findings, we integrated our own results with publicly available metadata. The CEA shows significant GABA_A_-α_2_ expression in both mice [38] (http://mouse.brain-map.org; cf. Figure 2k) and humans [39] (http://human.brain-map.org; Supplementary Fig. S10). Since amygdala modulation by BZDs occurs in humans (Supplementary Fig. S10) and PKCδ^+^ neurons may also exist in the human CEA [39] (http://human.brain-map.org, search term “PRKCD”), it is plausible that the proposed mechanism (Supplementary Fig. S9) is shared by humans.

Our data show that a specific behavioral effect of a broadly acting psychoactive drug converges on a specific circuit mechanism, a principle that may apply to other classes of psychoactive drugs. In addition to the neurons expressing the primary binding sites (here, GABA_A_-α_2_ receptor subunits on SST^+^/PKCδ^−^ neurons), the overlying circuit mechanism (here, inhibitory gating) in itself represents an interesting target for circuit directed therapies. Together, this study delineates an experimental strategy that integrates unbiased screening with increased preexisting understanding of circuit interactions underlying biomedically relevant behaviors. This approach permits probing circuit mechanisms mediating specific drug effects and evaluating their translational potential.

## Supplementary Information

Supplementary Information
