# Supplementary material for: Central amygdala circuit dynamics underlying the benzodiazepine anxiolytic effect
Source: Mol Psychiatry. 2018 Nov 30;26(2):534–44. doi: 10.1038/s41380-018-0310-3 (PMC6411154; doi:10.1038/s41380-018-0310-3)
Supplement: Supplementary file 1 — Supplementary Information [file 41380_2018_310_MOESM1_ESM.pdf]

Supplementary Information

Supplementary Methods

Subjects

Male C57BL/6J mice (2-6 months old) purchased from Charles River Laboratories (Sulzfeld, Germany) were used to assess limbic c-Fos expression (n = 68) and to test the interaction of DZP and PVT-CEAl projection using fMRI (n = 7). To test the involvement of PKCδ^{+}/SST^{-} neurons in the central lateral amygdala (CEAl), PKCδ::GluClα-CRE BAC transgenic mice (1) (PKCδ::Cre, mmrrc #11559, n = 38) were used. SST-IRES-Cre mice (SST::Cre, Jackson #013044) and PKCδ::Cre mice were crossed to Rosa::loxP-STOP-loxP-td-Tomato (Jackson #007905). The offspring (n = 4 for each cross) were used for neuronal population sequencing (n = 4 for each cross) and electrophysiological recordings (n = 8). For deep brain calcium imaging experiments, male SST-IRES-Cre mice (n = 3; for CEAl recordings), PKCδ::Cre mice (n = 4; for CEAl recordings) and C57BL6/J (n = 4; for CEAm recordings) mice were used. All transgenic animals were on a C57BL/6J background and heterozygous for the transgenic allele. Animals were genotyped after weaning and group-housed (2-5 animals/cage) at 21 °C with food and water provided ad libitum in a 14h light and 10h dark cycle (day starting at 6:00 a.m.). All tests were performed during the light period.

All animal experiments were performed in accordance with institutional guidelines and were approved by the respective Austrian (BGBl nr. 501/1988, idF BGBl I no. 162/2005) and European (Directive 86/609/EEC of 24 November 1986, European Community) authorities and covered by the license MA58/002220/2011/9.

Subject history

For the c-Fos screen, animals were exposed to the appropriate anxiogenic stimuli and euthanized. Their brains were prepared for histological assays. Home cage animals were taken from their home cage and euthanized directly. To test the involvement of PKCδ$^+$/SST$^-$ neurons in the central lateral amygdala, animals underwent surgery for virus injection, later tested first in the EPM and then in a reward-fear conditioning paradigm. After the last test, animals were euthanized and virus expression their brains was histologically assessed. For testing the interaction of DZP and PVT-CEAl projection using fMRI, animals underwent surgery for virus injection and fiber implantation and were then subjected to the fMRI scans. After the last test, animals were euthanized and virus expression in their brains was histologically assessed. For deep brain calcium imaging experiments, mice underwent surgery for virus injection and microendoscopic fiber implantation and were later fear conditioned and re-exposed to the aversive conditioning context. For neuronal population sequencing and electrophysiological recordings, mice were taken from their home cages and euthanized directly.

Stereotactic Surgery

Surgeries were performed using a Model 1900 Stereotactic Alignment Instrument (David Kopf Instruments) and a Model 1911 stereotactic drill (David Kopf Instruments). For injections, a Nanoliter 2000 injector, driven by a Micro4™ MicroSyringe Pump Controller (World Precision Instruments), was used. Needles for virus injection were pulled from 3.5nl glass capillaries (World Precision Instruments) on a Micropipette Puller (Model P-97, Sutter Instruments). The surgical protocol was adapted from Athos and Storm (2). Mice were deeply anaesthetized in the stereotactic frame with Isofluorane (1.7%, IsoFlo®, Abbot Laboratories) and anesthesia was verified by testing deep plantar reflexes. Gentamicin ointment (Refobacin® 3mg/g, Merck) was used to protect the animals' eyes, and their body temperature kept constant at 36°C using a heating pad. In order to selectively manipulate PKCδ$^+$ neurons in the CEAl, viral vectors (adeno-associated virus serotype 2/5, $AAV_{2/5}$) carrying double-floxed constructs were infused into the CEAl of PKCδ::GluClα-CRE BAC transgenic mice: $AAV_{2/5}$-EF1α-DIO-hM4D-mCherry-WPRE, $AAV_{2/5}$-hSyn-DIO-hM3D-mCherry-WPRE-hGh or

$AAV_{2/5}$-hSyn-eGFP-WPRE-hGh was injected into the CEAl bilaterally at a speed of 35 nl/min at the stereotactic coordinates from bregma: -1.36mm AP, ±2.9mm ML, 4.9mm DV. For calcium imaging, AAV_1-Syn-Flex-GCaMP6f-WPRE-SV40 was injected into the right CEAl of PKCδ::Cre or SST::Cre mice, at -1.36mm AP, 2.9mm ML, 4.9mm DV from bregma, and AAV_9-hSyn-GCaMP6m-WPRE was injected into the right CEAm at -1.06mm AP, 2.25mm ML, 4.6mm DV from bregma, both at a speed of 35 nl/min. In both cases, a microendoscopic fiber was implanted 200µm above the target structure. For stimulating PVT-CEAl projections in the fMRI, $AAV_{2/5}$-hSyn-hChR2(H134R)-eYFP-WPRE-hGh or $AAV_{2/5}$-hSyn-eGFP-WPRE-hGh was injected into the PVT at -1.58mmAP, 0mm ML, 3.4mm DV, and an optic fiber (Doric lenses, 240 µm, 0.22NA) was implanted over the right CEAl at -1.36mm AP, 2.85mm ML, 3.4mm DV). Dental cement (SuperBond C&B kit, Prestige Dental Products) was used to fix the fiber onto the skull.

When injections were finished, the needle was kept in place for an additional 5 min to minimize leakage when the needle was pulled out. The scalp was then closed with 2 to 3 stitches, and the animal placed into a clean cage by itself, where its body temperature was kept constant using a heating pad. Once the animal was awake, it was transferred back to its home cage, where it received enrofloxacin (100mg/ml, Baytril®, Bayer Austria) and carprofen (Rimadyl®, 50mg/ml; Pfizer Austria) via drinking water for 14 days. Animals were allowed to recuperate for at least 5 weeks before the first experiments to allow for optimal virus expression. For detailed virus information see Supplementary Table S1.

Pharmacology

Clozapine-n-oxide (CNO; Sigma-Aldrich Co. LLC) was dissolved in 1xPBS and used on the same day for intraperitoneal (i.p.) injection at 5 mg/kg. This dose was chosen to ensure effective silencing. CNO is metabolized to clozapine *in vivo*, potentially resulting in unwanted psychoactive effects mediated by clozapine. However, the clozapine level back-converted from CNO is quite low (a CNO:clozapine ratio of about 20:1 (3)), and CNO doses of up to 10 mg/kg do not affect locomotion behavior in the timeframe of the experiment (3). In our study, CNO treatment did not alter the total distance travelled or the time in the open arms of the EPM when compared to controls – the most critical behavioral parameters in the context of this study (cf. Fig. 4c,f, saline

GFP group and Supplementary Fig. S1a,d, saline group). However, to account for potential side effects, we chose an experimental design in which all groups received an equal dose of CNO. Diazepam (Gewacalm®, Nycomed Austria GmbH) was diluted in 1xPBS just before i.p. injection at 1 mg/kg. The injected volume was 5 ml/kg for each substance. Behavioral tests were performed 30 min after drug injection.

Behavioral Tests

Anxiety tests

A standard elevated plus maze (EPM) assay was used. The apparatus was raised 40 cm from the ground and consisted of two opposed open arms (35 x 5 cm) and two opposed closed arms (35 x 5 x 15 cm) linked by a square center zone (5 x 5 cm). Animals were placed individually in the center zone facing an open arm and allowed to explore the maze freely for 5 min. All anxiety tests were recorded and analyzed using ANY-maze (Stoelting Europe). Entries were counted when an animal's center point moved into the open arms.

For the c-Fos screen, 15 min after injection animals were placed in a novel chamber for 10 min where only the appropriate cohorts received 10 foot-shocks, 0.5mA, of 1s at randomized intervals of 20-100s. Mice were then transferred back to their home cage where they either stayed for 5 min before they were exposed to the EPM, or for 95 min until euthanasia.

Context fear conditioning

Fear conditioning occurred in 4 identical experimental chambers (16.5 cm wide x 16.5 cm deep x 30.5 cm high, H10-11M-TC, Coulbourn Instruments, Whitehall, PA, USA) encased in a sound-attenuating shell. Above the chamber, a custom-made house light provided illumination (around 10 lux), an infrared spotlight (Kemo Electronic, Geestland, Germany) improved animal detection, a speaker (Audiocomm, Vienna, Austria) provided sounds designed using Audacity software (http://www.audacityteam.org) with a maximal sampling frequency of 192 kHz and played from a Terratec sound card (Alsdorf / Germany). A video camera (Basler, Ahrensburg, Germany) monitored the animal's behavior. Shocks were delivered via a stainless-steel shock floor

(H10-11M-TC-SF, Coulbourn Instruments), and chambers were cleaned with diluted, lemon-scented cleaning solution before each mouse. The house lights were turned off. The fear conditioning session started with a 2 min baseline, followed by conditioned stimulus (CS) presentation (3 kHz tone, total duration of 10 s, 75 dB) which was again immediately followed by a foot shock (1 s, 0.5 mA) delivered to the floor via an external shocker (H13-15, Coulbourn Instruments). The session lasted 10.5 min and consisted of 5 trials with a randomized ITI of 100 ± 30 s.

Freezing was scored with Ethovision XT 8 (Noldus Information Technology, Wageningen, the Netherlands) and defined as a lack of all movement except for respiratory-related movements (4). To control the success of fear conditioning, freezing to each aversive-CS presentation was compared to a 2-min baseline period. Videos were recorded at a rate of 20 frames/s and analyzed with Ethovision using an immobility threshold of 2.5% pixel change per sample and sampling of 0.5 s (determined by eye, blind to experimental conditions). For calcium imaging, mice were re-exposed to the conditioning context on two separate days. Before each session, mice received i.p. injections of either DZP (1 mg/kg, day 8) or saline (day 10). When comparing baseline freezing levels from day 1 (before conditioning) to freezing in the fear context (day 10), mice showed increased freezing to the context (mean ± SEM: baseline = 3.17±2.06%, context = 23.48±5.97%; n = 11 mice; paired t-test: t = 3.448, df = 10, P = 0.03).

Histology and Immunohistochemistry

To assess c-Fos or virus expression, mice were deeply anesthetized and perfused transcardially with 10 ml of 10U Heparin/ml PBS and 30 ml of 4% paraformaldehyde. In the case of c-Fos expression, perfusions were timed to exactly 90 min after completion of behavioral tests or 120 min after i.p. injection. For all animals, brains were extracted and left overnight in 30% sucrose (wt/vol) at 4°C. The following morning, they were transferred to a 1:1 mixture of 30% sucrose and Tissue Tech ® O.C.T., followed by a transfer to plain O.C.T. that evening, and subsequently frozen in O.C.T on dry ice and stored at -80°C until sectioning. Coronal cryosections were cut at 20 μm thickness. Sections were stained with primary antibody at 4°C overnight in a blocking solution containing 1% BSA and 0.1% Triton X-100 (vol/vol). After four 10 min washes, standard Alexa Fluor secondary antibodies (Thermo Fisher Scientific, 1:1000; A11031 for PKCδ, and A11008 for c-Fos) and DAPI were used at room temperature for 2 h. Sections were then washed six times for 10 min each and mounted in Fluorescence Mounting Medium (Dako, S3023). Primary antibodies used: mouse antibody to PKCδ (BD Biosciences, 610398 1:1000), rabbit antibody to c-Fos (Abcam, ab 7963, 1:1000) and rabbit antibody to DsRed (Living Colors, 632496; 1:1000; to optimize visualization of AAV::DIO-M3 and AAV::DIO-M4 expression, both tagged with mCherry fluorophore). Whole slides were then scanned using an automated widefield-microscope (Pannoramic 250 Flash, 3D HISTECH Ltd.). Once images were acquired, regions of interest were marked by hand (5) within Panoramic Viewer (3D HISTECH Ltd.). Each animal needed at least 2 samples of each region for inclusion in the analysis. For Fig. 2a-h, each animal had at least 3 samples for CEAl. In general, experimental groups had samples from at least 3 different animals for each region, except for the following combinations which consisted of samples from only 2 animals: the bed nucleus of the stria terminalis (BST), the ILA, the prelimbic area (PL) and the rostroventral part of the lateral septal nucleus (LSr). These were all in the 'no injection' (homecage) group and only used for illustration in Fig. 1b, but not for any further analysis. Please see Table S4 for details. To assess virus spread, mean fluorescence was quantified using Fiji ImageJ (6). A Zeiss LSM 710 Spectral confocal microscope was used to produce exemplary scans for

figures. See Table S3 for qualitative assessment of virus expression. Representative examples and quantification of virus expression are shown in Fig. S7 (chemogenetic manipulations) and Fig. S8 (optogenetic manipulation).

Cell Counting

For analyzing images, a semi-automated, machine learning based approach was used. After brain regions were first marked in Pannoramic Viewer, they were exported and analyzed in Definiens Developer XD. First, the nuclei were segmented on DAPI using a LoG-Filter and watershed algorithms. On a set of training images, nuclei objects were manually classified as positive and negative and used as samples for training a machine learning algorithm. Mean intensity, standard deviation and local contrast parameters were extracted from these as input for a decision tree based classifier. This classifier was then applied to all nuclei objects on a larger set of images to define positive cells. To assess nuclear and cytoplasmic co-labeling, images were hand-counted by a blinded investigator. Raw c-Fos numbers obtained with this staining and quantification protocol (CEA ~ 4 – 60 / mm^2, BLA ~ 25 – 100 / mm^2, IL ~ 0 – 250 / mm^2) were in the typical range for c-Fos IHC studies (7-10). To compare c-Fos expression levels, the number of c-Fos positive nuclei was then normalized to the number of DAPI positive nuclei in any given region.

c-Fos Network Analysis

The functional network analysis was performed using an in-house application implemented in Python 3 and a series of open source libraries available at http://dx.doi.org/10.5281/zenodo.60880 under the GPLv3 License. Input to the application consists of connectome data describing the structural connectivity of the studied regions, c-Fos expression data under different treatment conditions, a consistent name mapping scheme for region names in the connectome and the c-Fos data, and a configuration file that guides the data processing and algorithm execution.

First, the c-Fos expression data (containing multiple samples per region and mouse) was loaded and aggregated by first calculating the mean expression over each region per mouse, followed by a mean over all mice for each

region. This procedure produced one average expression value per region and treatment group, which were then converted to z-scores for each region over all treatment groups: one for all groups including the control group (i.e. home cage mice) and one without the control group. The z-scored data containing the no-injection (i.e. home-cage) control group was used solely to calculate hierarchical clustering using Euclidian distance and the average linkage clustering agglomeration method. The data without the no-injection group were used for further analysis.

Second, the c-Fos data were used to compute correlation matrices describing the correlation between expression rates of two brain regions under with-drug and without-drug treatment conditions (Fig. S3a,b). Without-drug treatment conditions included 'saline', 'saline & EPM', 'saline & shock', 'saline & shock &EPM' while the with-drug treatment conditions are 'DZP', 'DZP & EPM', 'DZP & shock', 'DZP & shock & EPM'. The calculation was performed by building a vector of 4 z-scored expression values (one for each treatment condition) for the with-drug/without-drug treatment conditions and calculating their Pearson correlation (ρ).

Third, φ was calculated by taking the c-Fos correlation values calculated in the second step and calculating the sum of all pairwise correlations with each region ($\varphi = \Sigma\rho$). The effect of the drug (DZP) was analyzed by calculating the difference of φ between drug and no drug measures (i.e. $\Delta\varphi = \varphi_{DZP} - \varphi_{saline}$). Fourth, the connectome was imported from the Allen Mouse Brain Atlas, whereby recursive projections were filtered out, region names were mapped according to user specification, and multiple projections between the same two regions were aggregated by taking their mean. We used the left-brain projections from the Allen Mouse Brain Connectivity Atlas. From the connectome, a subset of brain regions was extracted that contains only regions present in the c-Fos expression data. This connectome subset was then normalized to the outgoing edges of each region ($\Sigma\zeta_{out} = 1$, ζ_{out} being the connection strength of outgoing connections).

Next, φ_{con} was calculated by taking the c-Fos correlation values calculated in the second step and scaling them by the normalized connectome generated in the third step. In order to obtain a single measure for each node in the network, the sum of all scaled correlation pairs was calculated from that node to its first order neighbors $\varphi_{con} = \Sigma\rho\zeta$. This then served as a proxy, combining functional and structural information as well as reflecting

the change in correlation between two regions. The effect of the drug (DZP) is analyzed by calculating the difference of φ_{con} between drug and no drug measures (i.e. $\Delta\varphi_{con} = \varphi_{con(DZP)} - \varphi_{con(saline)}$). As φ_{con} is a network measure on a directed graph, it is possible to perform a node analysis only based on outgoing edges (i.e. φ_{con} Output) or incoming edges (i.e. φ_{con} Input). A total of 62 animals were used for the correlation analysis over all conditions and treatments, resulting in the sampling from ACAd $n_{saline} = 24$, $n_{DZP} = 22$; ACAv $n_{saline} = 23$, $n_{DZP} = 22$; AI $n_{saline} = 29$, $n_{DZP} = 25$; BLA $n_{saline} = 27$, $n_{DZP} = 24$; BST $n_{saline} = 19$, $n_{DZP} = 18$; CEA $n_{saline} = 30$, $n_{DZP} = 24$; ILA $n_{saline} = 18$, $n_{DZP} = 20$; LA $n_{saline} = 27$, $n_{DZP} = 24$; LSc $n_{saline} = 23$, $n_{DZP} = 21$; LSr $n_{saline} = 23$, $n_{DZP} = 21$; LSv $n_{saline} = 22$, $n_{DZP} = 21$; PAG $n_{saline} = 26$, $n_{DZP} = 23$; PL $n_{saline} = 18$, $n_{DZP} = 20$; PVH $n_{saline} = 23$, $n_{DZP} = 24$; PVT $n_{saline} = 30$, $n_{DZP} = 28$.

Neuronal population sequencing

PKCδ::Cre or SST::Cre mice were crossed to Rosa::loxP-STOP-loxP-td-Tomato animals, and the offspring used for neural population sequencing. Males (2-5 months old) were decapitated, brains extracted on ice and 1mm thick brain sections cut in ice-cold Hibernate A Low Fluorescence solution (BrainBits). The central amygdala was extracted using biopsy punchers (1mm diameter; Integra Miltex) and enzymatically dissociated (Papain Dissociation System, Worthington Biochem). Approximately 10^3 td-Tomato$^+$ cells were retrieved with FACS sorting. For each animal, the central amygdala was extracted once on each side and then pooled.

Libraries were prepared with the SMARTer® smRNA-Seq Kit for Illumina® (Clontech, 78100 Saint-Germain-en-Laye, France) and submitted to deep sequencing on a HiSeq 2500 system (Illumina, San Diego, USA). Reads aligning to rRNA sequences were filtered out with BWA against RefSeq rRNAs (11). Transcript per million (TPM) values were calculated with Kallisto (12) (v0.43.0) using UCSC Genome Browser RefSeq annotation (downloaded on 20 February 2015). Standard parameters were used, and fragment length was set to 200 nucleotides.

Ex vivo electrophysiology

To prepare brain slices, 2-3 month old male SST::tdTomato mice were deeply anesthetized with Isoflurane, decapitated and their brains submerged in ice-cold dissection buffer (220mM sucrose, 26mM $NaHCO_3$, 2.4mM KCl, 10mM $MgSO_4$, 0.5mM $CaCl_2$, 3mM sodium pyruvate, 5mM sodium ascorbate and 10mM glucose), gas-flushed with 95% O_2/5% CO_2. 300µm thick brain slices were cut in dissection buffer with the help of a vibratome (Leica, VT1000S) and transferred to oxygenated aCSF in 95% O_2/5% CO_2 at 32°C for a 15 min recovery step. After that, slices were stored in oxygenated aCSF (126mM NaCl, 2.5mM KCl, 1.25mM NaH_2PO_4, 26mM $NaHCO_3$, 2.5mM $CaCl_2$, 2.5mM $MgCl_2$, and 25mM glucose) at room temperature, at least 30 min before recording.

Individual brain slices were visualized with infrared-differential interference contrast microscopy (Olympus BX50WI) and digitized with an infrared sensitive digital camera (Hamamatsu, ORCA-03). Slices were entirely submerged and continuously perfused at a rate of 1-2 ml per min with oxygenated aCSF. SST^+ / SST^- neurons in the CEl were identified by expression of tdTomato. Patch pipettes were pulled to final resistances ranging from 3 to 5MΩ (Sutter, P-97). Membrane currents were recorded (Multiclamp 700B amplifier; Molecular Devices) and signals low-pass filtered at 3kHz, sampled at 10kHz and analyzed with pClamp 10 software.

The cell was allowed to reestablish constant activity post break-in for 5 minutes and a baseline was recorded for at least 5 minutes in whole-cell voltage-clamp configuration (-70mV), followed by application of Diazepam (10µM) to the bath. Frequency and shape of spontaneous inhibitory post-synaptic currents (sIPSCs) were analyzed using Clampfit software (Molecular devices). Two min bins of sIPSCs from before and after addition of diazepam were analyzed for each cell. Internal solution contained the following: 130mM KCl, 10mM HEPES, 10mM EGTA, 5mM $CaCl_2$, 2mM $MgCl_2$, 2mM Na_2ATP, 0.4mM NaGTP, 5mM Na_2Phosphocreatine.

Statistical analysis.

Sample size. The sample size for all experiments was based on previous experiments and published literature (13, 14).

Exclusion of data points. Virus-injected mice that did not show virus expression in post-mortem histological classification were excluded from all data analyses (see Supplementary Table S3). Further, one mouse (saline M4 cohort) had to be excluded from the analysis of the DREADD EPM experiment because it jumped off the apparatus. These routine exclusion criteria had been established prior to testing.

Randomization. Within each experiment, animals were assigned to treatment groups at random, and assignment was balanced over cages.

Blinding. For the c-Fos analysis, cell counting was either performed by an experimenter blinded to the experimental conditions or computationally. For slice electrophysiology, the experimenter was not blinded to drug treatment. For deep brain calcium imaging experiments, the experimenters were not blinded to drug treatment, but analysis was performed computationally. For behavioral experiments, the experimenter was not blinded, but analysis was performed blinded or computationally. For fMRI experiments, the experimenter was not blinded to drug treatment, but was blinded to viral expression.

Normality. For c-Fos, deep brain calcium imaging and gene expression data, normality of the underlying populations was assumed. *Ex-vivo* electrophysiology, mouse behavior, and virus expression data were tested for normality using D'Agostino & Pearson normality test. If normality was not detected, non-parametric statistical tests were used. For fMRI, standard statistical workflows were used, and data were treated as normally distributed.

Variation. Variance is represented by individual data points in each graph. Corrections for unequal variances were applied whenever possible.

Unless indicated otherwise, all statistical analyses were performed in Graph Pad Prism ® (Version 7). The statistical tests used are indicated in the figures legends, two-tailed, and were corrected for multiple comparisons whenever applicable. Wherever significance is not made explicit, the test did not reach statistical significance.

fMRI literature search

To find brain regions involved in the effect of BZD on fear and anxiety, PubMed (https://www.ncbi.nlm.nih.gov/pubmed/) and the Cochrane Library (http://www.cochranelibrary.com) were searched (October 2016, search terms below). Results not relevant for fear or anxiety were excluded. From the remaining literature (see Table S2), a meta score for each region was calculated as the sum of all region-specific BZD effects found in this literature set. Regions were rank ordered based on that score.

Search terms

PubMed: (benzodiazepine[Title/Abstract] OR Adinazolam[Title/Abstract] OR Alprazolam[Title/Abstract] OR Bentazepam[Title/Abstract] OR Bretazenil[Title/Abstract] OR Bromazepam[Title/Abstract] OR Brotizolam[Title/Abstract] OR Camazepam[Title/Abstract] OR Chlordiazepoxide[Title/Abstract] OR Cinazepam[Title/Abstract] OR Cinolazepam[Title/Abstract] OR Clobazam[Title/Abstract] OR Clonazepam[Title/Abstract] OR Clonazolam[Title/Abstract] OR Clorazepate[Title/Abstract] OR Clotiazepam[Title/Abstract] OR Cloxazolam[Title/Abstract] OR Delorazepam[Title/Abstract] OR Diazepam[Title/Abstract] OR Diclazepam[Title/Abstract] OR Estazolam[Title/Abstract] OR Etizolam[Title/Abstract] OR Ethyl loflazepate[Title/Abstract] OR Flubromazepam[Title/Abstract] OR Flubromazolam[Title/Abstract] OR Flunitrazepam[Title/Abstract] OR Flurazepam[Title/Abstract] OR Flutazolam[Title/Abstract] OR Flutoprazepam[Title/Abstract] OR Halazepam[Title/Abstract] OR Ketazolam[Title/Abstract] OR Loprazolam[Title/Abstract] OR Lorazepam[Title/Abstract] OR Lormetazepam[Title/Abstract] OR Medazepam[Title/Abstract] OR Mexazolam[Title/Abstract] OR Midazolam[Title/Abstract] OR Nifoxipam[Title/Abstract] OR Nimetazepam[Title/Abstract] OR Nitrazepam[Title/Abstract] OR Nordiazepam[Title/Abstract] OR Oxazepam[Title/Abstract] OR Phenazepam[Title/Abstract] OR Pinazepam[Title/Abstract] OR Prazepam[Title/Abstract] OR Premazepam[Title/Abstract] OR Pyrazolam[Title/Abstract] OR Quazepam[Title/Abstract] OR Rilmazafone[Title/Abstract] OR Temazepam[Title/Abstract] OR Tetrazepam[Title/Abstract] OR Triazolam[Title/Abstract] OR Flumazenil[Title/Abstract] OR Eszopiclone[Title/Abstract] OR Zaleplon[Title/Abstract] OR Zolpidem[Title/Abstract] OR ("zopiclone"[Supplementary Concept] OR "zopiclone"[All Fields])) AND (fMRI[Title/Abstract] OR functional imaging[Title/Abstract] OR functional neuroimaging[Title/Abstract] OR neuroimaging[Title/Abstract] OR BOLD[Title/Abstract] OR ("magnetic resonance imaging"[MeSH Terms] OR ("magnetic"[All Fields] AND "resonance"[All Fields] AND "imaging"[All Fields]) OR "magnetic resonance imaging"[All Fields] OR ("functional"[All Fields] AND "mri"[All Fields]) OR "functional mri"[All Fields])) AND human[Title/Abstract].

Cochrane library: (benzodiazepine OR Adinazolam OR Alprazolam OR Bentazepam OR Bretazenil OR Bromazepam OR Brotizolam OR Camazepam OR Chlordiazepoxide OR Cinazepam OR Cinolazepam OR Clobazam OR Clonazepam OR Clonazolam OR Clorazepate OR Clotiazepam OR Cloxazolam OR Delorazepam OR Diazepam OR Diclazepam OR Estazolam OR carfluzepate

OR Ethyl carfluzepate OR Etizolam OR Ethyl loflazepate OR Flubromazepam OR Flubromazolam OR Flunitrazepam OR Flurazepam OR Flutazolam OR Flutoprazepam OR Halazepam OR Ketazolam OR Loprazolam OR Lorazepam OR Lormetazepam OR Medazepam OR Mexazolam OR Midazolam OR Nifoxipam OR Nimetazepam OR Nitrazepam OR Nordiazepam OR Oxazepam OR Phenazepam OR Pinazepam OR Prazepam OR Premazepam OR Pyrazolam OR Quazepam OR Rilmazafone OR Temazepam OR Thienalprazolam OR Tetrazepam OR Triazolam OR Flumazenil OR Eszopiclone OR Zaleplon OR Zolpidem OR Zopiclone) AND (fMRI OR functional imaging OR functional neuroimaging OR neuroimaging OR BOLD OR functional MRI) AND human.

fMRI experiments and analysis

2 month old male animals, virally expressing ChR2 in the PVT and with optogenetic fiber implants above the CEAl (Supplementary Fig. S5a) were slightly anesthetized by isoflurane (~1.2 % resulting in ca. 60 respiration beats per minute). This low dose Isoflurane, a frequently used anaesthetic agent in rodent fMRI (15), leads to the most reproducible activation patterns (intra-individual as well as inter-individual) compared to a variety of other agents as shown by Schroeter et al. in a recent study (16). At the dorsal surface of the right hind paw a customized feed-back controlled Peltier element (Kryotherm, Saint-Petersburg, Russia) was placed. A remote-controlled laser was fiber-optically attached to the implanted optical port. Both stimulation devices were triggered by the MR via a custom-made stimulation program (LabView). 30 min prior to the MRI session, the animals were injected i.p. with ~150 µl injection volume of 1 mg/kg Diazepam (Gewacalm®, Nycomed Austria GmbH) in PBS or PBS only. Supplementary Fig. S5 gives a graphical summary of the analysis workflow described below. The stimulus protocol consisted of 8 repetitions of a series of a heat stimulation (15 s ramp and 5 s plateau of 50 °C heat pulses), a laser stimulation (20 s 10 mW 20 ms pulses of 478 nm light delivered at 10 Hz) and 20 s of a combined heat + laser stimulations again followed by the laser stimulation. All stimuli were separated by a 1 min inter-trial interval (Supplementary Fig. S5b). The heat stimuli were used to drive a functional aversive – nociceptive – network of activated brain structures.

Functional BOLD MRI scans were performed using a T2*-weighted single-shot gradient echo based EPI. A functional scan consisted of 975 acquisitions of 22 axial slices (EPI, 64 x 64 matrix, TR = 2000 ms, averaging of 2, resulting in total TR of 4000 ms (needed for covering 22 slices), TEef = 24.4 ms, FOV 15 x 15 mm, in-

plane spatial resolution 234 x 234 μm, slice thickness 500 μm). Next, an anatomical reference scan was performed at slice positions identical to the functional ones by a fast spin echo sequence (RARE, 256 x 256 matrix, TR= 3000 ms, RARE factor= 8, TEef= 47.1 ms, FOV 15 x 15 mm, NEX= 10, slice thickness 500 μm). FMRI raw data were pre-processed by motion correction using rigid registration and resampling with sinc interpolation, slice time correction performed with a Cubic Spline interpolation, linear detrending, high pass filtering (9 cycles) and temporal smoothing (12 seconds) and spatial 2D Gaussian smoothing of the data (FWHM kernel: 2 pixel, in-plane direction). After pre-processing, we conducted a GLM analysis with the different stimulation condition as separate predictors (Supplementary Fig. S5b) using BrainVoyager (QX 2.8, Brain Innovation B.V. Maastricht, The Netherlands), applying a cluster threshold of 4 voxels. The single Statistical Parametric Maps (SPM), obtained for each animal and predictor, were labelled using a lab customized 3D mouse brain atlas derived from Paxinos et al. (5) resulting in 196 identified brain structures (Supplementary Fig. S6a). The following detailed graph-theoretical connectivity analysis was performed on focussing on the laser stimulation predictor only to investigate the interaction of optogenetic activation and drug treatment. Consequently, the average time courses of the voxels (as percentage BOLD signal change) of each brain structure, significantly activated by the laser stimulation (Supplementary Fig. S5c, blue line), were corrected for global signal fluctuations by linear regression. The residual average time courses of all brain structures (Supplementary Fig. S5d) were cross-correlated for the laser predictor and each animal. Next, the individual cross-correlation matrices were averaged over all animals per group resulting in averaged cross-correlation matrices (Supplementary Fig. S5e). In order to compare the number of connections across stimulation condition and experimental groups the average matrices were binarized using FDR threshold ($\alpha \leq 0.05$). These binarized matrices were used to create networks (i.e. graphs). These graphs were visualized in a custom-made module in Amira based on the 3D coordinates of each brain structure – node – taken from our 3D mouse brain atlas derived from Paxinos et al. (5). Nodes without edges (orphan nodes) were omitted. The node-size represents the sum of edges, i.e. significant connections with all other brain structures. For a better appreciation of the 3D node distribution, a transparent mouse brain surface of an average anatomical standard

mouse brain was rendered and used as anatomical reference. Only the nodes of the limbic subnet, guided by the c-Fos data (amygdala hip area, amygdala piriform, anterior amygdala, basolateral amygdaloid nucleus, basomedial amygdaloid nucleus, bed nucleus of stria terminalis, central nucleus of the amygdala, cingulate cortex, cortical amygdala, infralimbic cortex, insular cortex, medial amygdaloid nucleus, paraventricular hypothalamic nucleus, periaqueductal gray, prelimbic cortex, septum, sublenticular extended amygdala) were colour coded (Supplementary Fig. S6b).

To emphasize the differences of the experimental conditions to the control condition “GFP saline”, the average limbic subnet matrix of GFP saline was subtracted from the average limbic subnet matrices of the other groups resulting in networks with differences in functional connectivity strength as edge weight between the nodes. These difference subnets were displayed using a forced based projection algorithm (Kamadai-Kawa in NWB, NWB Team, 2006). Thus, nodes sharing edges, here differences in functional connectivity, with each other are clustered together (Supplementary Fig. S6e). The node-size represents the sum of the absolute differences of all edges of that node. The thickness of each edge codes its weight, the absolute difference in connectivity strength. Red edges indicate an increase of connectivity strength compared to GFP saline and blue edges a decrease (Fig. S6e). The colour of each node was chosen anatomically (see Supplementary Fig. S6b). Additionally, we took advantage of the non-invasive and high 3D resolution of the MRI modality for spatially referencing the placement of the optical fiber. Therefore, we perfused the animals under deep ketamine/xylazine anesthesia (100mg/10mg i.p.; Ketasol, Graeub Veterinary products/ Rompun®, Bayer), transcardially using 4% paraformaldehyde solution (PFA) made in 0.1M phosphate buffered saline (PBS) adding Prohance® (0,5 M, Braco Imaging, 1:9 mixture with PFA solution). The mouse head was stripped of unnecessary tissue and was carefully positioned in 10ml Falcon tube filled with fluorine based liquid Fluorinet FC-770 (3M company). In order to obtain a high-resolution anatomical reference for the fiber position 3D T1 weighted FLASH sequence was performed with TE=11.25 ms, TR=34.024 ms, flip-angle=20 degree, and 13 averages resulting in 6 h and 17 min scan time. The FOV was 20x16x16 mm and the corresponding matrix size 400x320x160 resulting in an isotropic resolution of 50x50x100 μm. Within the Amira program the fiber tip in each animal was marked in

3D and the corresponding coordinates used for depicting the fiber positions across the animals (cf. Supplementary Fig. S8f,g**).**

Deep brain calcium imaging

Deep brain calcium imaging was performed using a Vista HD 2.0 in vivo Rodent Brain Imaging System (Inscopix, Palo Alto, USA), with GLP-0561 microendoscopic fibers implants on GCaMP expressing animals (see Surgery). The animals were habituated to the imaging device with a dummy microscope for 1h. Behavioral control, Ca^{2+} and behavioral videos were recorded on a fully synchronized custom built setup, running on Anymaze, (Stoelting, Wood Dale, USA), Arduino 2.0 scripts and nVistaHD v2.0.32 software, respectively. Behavioral data was analyzed in Anymaze. Calcium imaging data was acquired at 20 fps and processed in the MOSAIC v1.2 software (Inscopix, Palo Alto, USA) und custom ImageJ scripts. Movies from the two recording sessions were motion compensated by a combination of automated registration (MOSAIC v1.2) and affine transformation of visually selected landmarks (custom ImageJ scripts). Here, the saline session recorded at the end of the experiment served as registration template. This resulted in motion-corrected movies covering both experimental session and allowing for reliable registration and observation of identified neurons throughout the experiment. Movies were smoothened by re-sampling with 1 x 4 spatial x temporal binning. Units were extracted by session-wise $\Delta F/F_0$ normalized recordings to compensate for drifts in recording conditions by PCA/ICA (MOSAIC v1.2) performed across all recording sessions. These automatically identified units were visually curated for quality and stability, yielding stably registered and identified units across both recording sessions. Traces from these units were low-pass filtered at 0.5 Hz (Fig. 3b, right, bottom). Calcium events were detected at a peak threshold > 6 S.D and decay time $\tau > 0.5$s. (Fig. 3b, right, top). Units were projected onto mean movies (Fig. 3b, left). Neuronal activity was expressed as events score, computed as the cumulative amplitude of above threshold calcium events for each neuron in each session (Fig. 3c, top). With this workflow, we analyzed neurons from a total of 3 animals (CEAl SST^{+} /$PKC\delta^{-}$), 4 animals (CEAl $PKC\delta^{+}$/SST^{-}) and 4 animals (CEm), yielding a total 24 units from 2 (CEAl SST^{+} /$PKC\delta^{-}$), 14 units from 3 animals (CEAl PKCδ

$^{+}$/SST^{-}) and 33 units from 4 animals (CEm) with significant activity (passing PCA/ICA detection). Population activity was expressed as average of the cell-wise event scores for each session. These units were classified based on d activity BZD-activity saline session in cells with increasing ($\Delta_{\text{event score BZD-saline}}$ >0), decreasing ($\Delta_{\text{event score BZD-saline}}$ <0) or unaffected ($\Delta_{\text{event score BZD-saline}}$ =0) activity, respectively (Fig. 3c, bottom).

Data availability

Neural population sequencing data (record GSE95154) are available at https://www.ncbi.nlm.nih.gov/geo/query/acc.cgi?token=oretwkoofbeppmp&acc=GSE95154.

Code availability

The code used for the c-Fos network analysis is available at http://dx.doi.org/10.5281/zenodo.60880 under the GPLv3 License.

Supplementary Figures

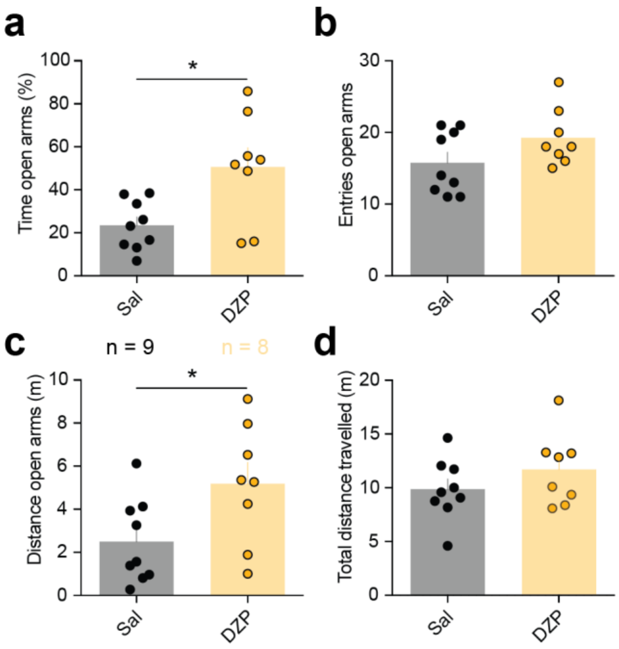

Supplementary Fig. S1 The chosen dose of DZP is anxiolytic but not sedative.
Mice were injected with either saline or DZP (1 mg/kg) and placed in an elevated plus maze for 5 min. **a** DZP at this dose had a clear anxiolytic effect as shown by the time spent in the open arms (unpaired t-test, $t = 2.923$, $df = 15$, $P = 0.0105$). **b** The entries into the open arms remained unchanged (unpaired t-test, $t = 1.7$, $df = 15$, $P = 0.1098$). **c** The distance travelled in the open arms increased (unpaired t-test, $t = 2.316$, $df = 15$, $P = 0.0351$). **d** The total distance travelled was not changed (unpaired t-test, $t = 1.218$, $df = 15$, $P = 0.2422$). Bars are means ± s.e.m. Significance levels between groups at * $P < 0.05$.

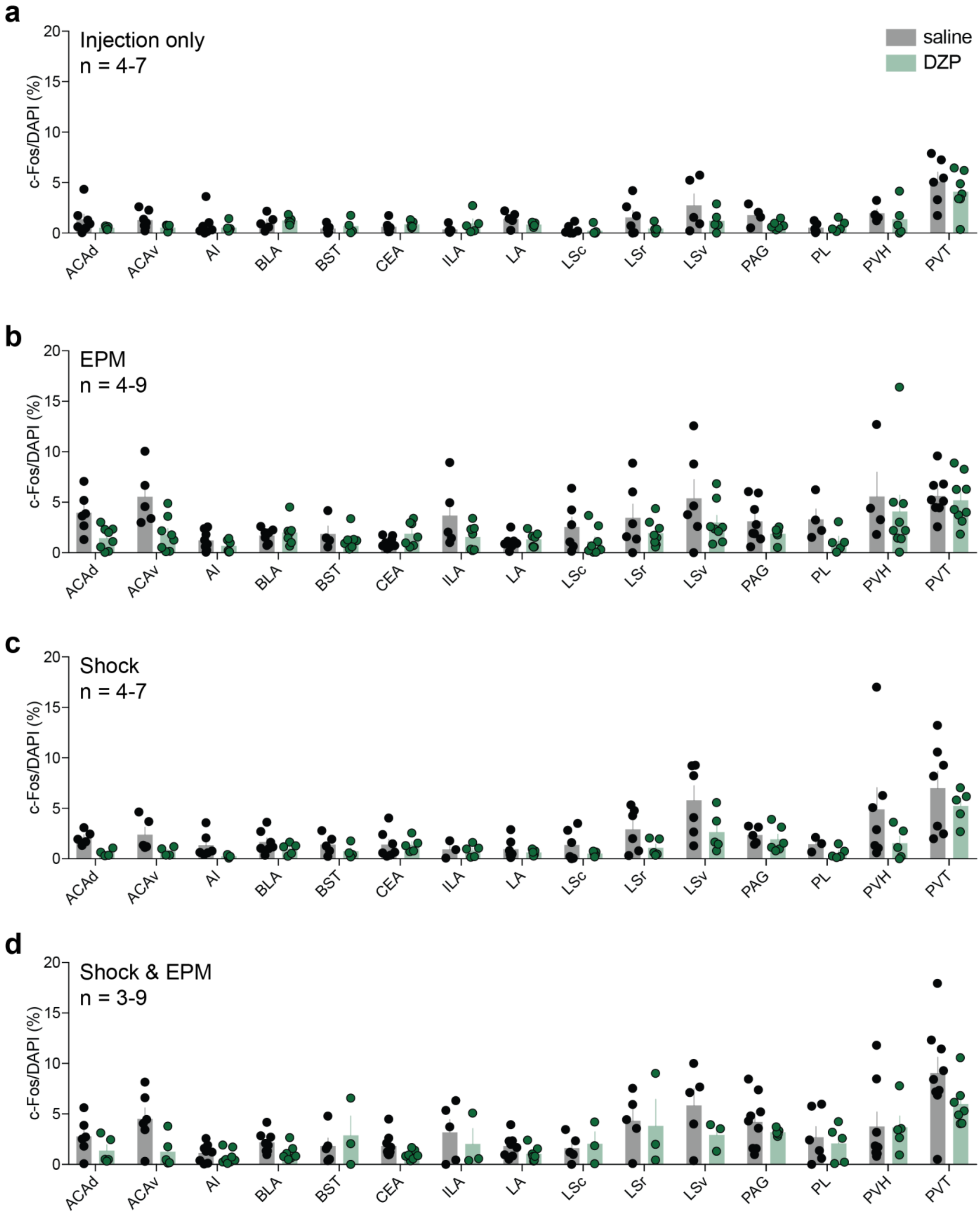
a
Injection only
n = 4-7
saline
DZP
b
EPM
n = 4-9
c
Shock
n = 4-7
d
Shock & EPM
n = 3-9
c-Fos/DAPI (%)
0
5
10
15
20
ACAd
ACAv
AI
BLA
BST
CEA
ILA
LA
LSc
LSr
LSv
PAG
PL
PVH
PVT

Supplementary Fig. S2 c-Fos expression in the limbic system in different anxiety states.
a-d DZP had an overall effect in each of the anxiety states tested. **a** Injection only cohort (two-way ANOVA $F_{interaction}$ (14, 131) = 0.7982, P = 0.6697; F_{region} (14, 131) = 10.53, P < 0.0001; F_{drug} (1, 131) = 5.651, P = 0.0189). **b** EPM cohort (two-way ANOVA $F_{interaction}$ (14, 172) = 1.074, P = 0.3840; F_{region} (14, 172) = 5.207, P < 0.0001; F_{drug} (1, 172) = 15.76, P = 0.0001). **c** Shock cohort (two-way ANOVA $F_{interaction}$ (14, 128) = 0.668, P = 0.8017; F_{region} (14, 128) = 6.146, P < 0.0001; F_{drug} (1, 128) = 13.66, P = 0.0003). **d** Shock & EPM cohort (two-way ANOVA $F_{interaction}$ (14, 148) = 0.7139, P = 0.7580; F_{region} (14, 148) = 7.285, P < 0.0001; F_{drug} (1, 148) = 7.851, P = 0.0058). Please see Table S4 for exact number of samples.

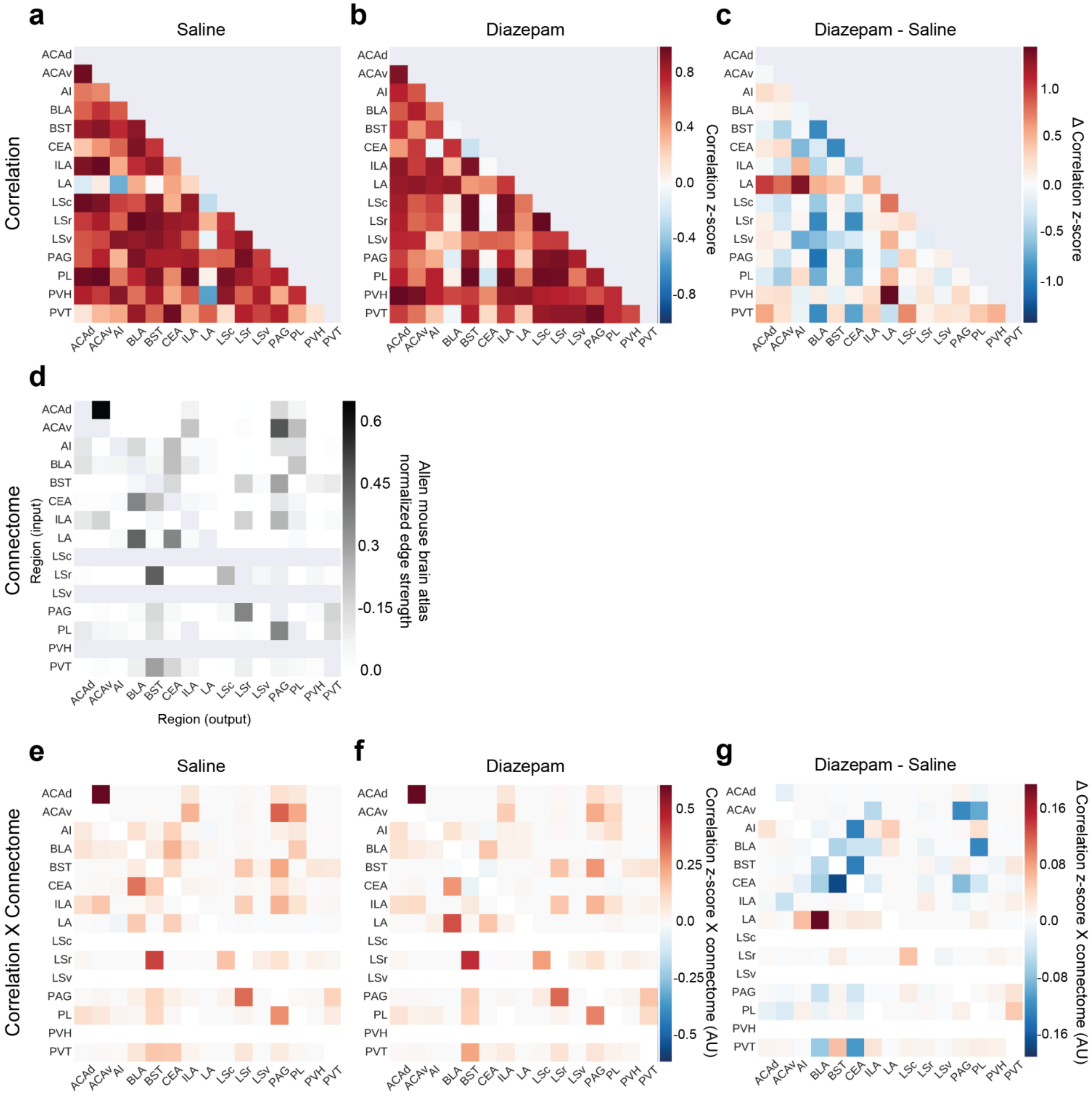

Supplementary Fig. S3 Functional and structural connectivity of a limbic network.
a-c Matrices showing the Pearson correlation in c-Fos levels between individual regions in the saline **a** and DZP states **b**, as well as the difference between DZP and saline states. The sum of rows and columns for any region corresponds to φ (**a,b**) or Δφ (**c**) described in Fig. 1. Values are z-scored group means calculated for each region over all drug and no-drug states. **d** Matrix showing the anatomical connections between regions, normalized to all outgoing connections for each region. Outgoing directionality is from rows to columns. **e-g** Multiplication of the connectome with the corresponding matrices from **a-c**. The sum of rows or columns corresponds to φ_{con} (**e,f**) or $\Delta\varphi_{con}$ (**g**) for input or output connections, respectively. ACAd – anterior cingulate area, dorsal part; ACAv – anterior cingulate area, ventral part; AI – agranular insular area; BLA – basolateral amygdalar nucleus; BST – bed nuclei of the stria terminalis; CEA – central amygdalar nucleus; ILA – infralimbic area; LA – lateral amygdalar nucleus; LSc – lateral septal nucleus, caudodorsal part; LSr – lateral septal nucleus, rostroventral part; LSv – lateral septal nucleus, ventral part; PAG – periaqueductal gray; PL – prelimbic area; PVH – paraventricular hypothalamic nucleus; PVT – paraventricular nucleus of the thalamus

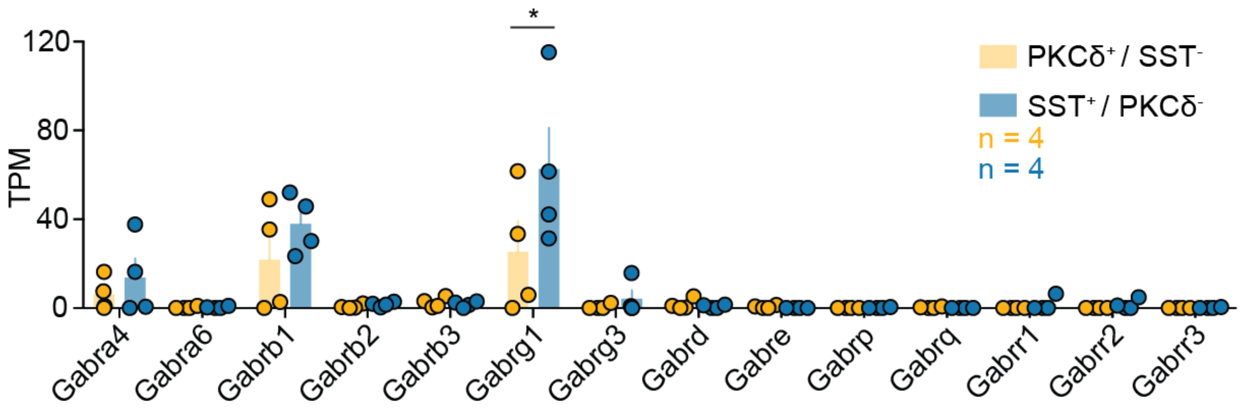

Supplementary Fig. S4 Expression of $GABA_A$ receptor subunits not strongly involved in BZD binding in CEA neuronal populations. Expression levels (transcripts per million reads mapped, TPM) of $GABA_A$-receptor subunits. The γ_1 subunit of the $GABA_A$ receptor is more strongly expressed in $SST^+/PKC\delta^-$ cells (RM two-way ANOVA $F_{interaction}$ (13, 78) = 1.957, P = 0.0359; F_{gene} (13, 78) = 12.83, P < 0.0001; $F_{cell\ type}$ (1, 6) = 2.371, P = 0.1745); Two-stage linear step-up procedure of Benjamini, Krieger and Yekutieli). Bars are means ± s.e.m. Significance levels between groups at * Q < 0.05. $GABA_A$ receptor subunits: Gabra4,6 – $\alpha_{4,6}$, Gabrb1-3 – $\beta_{1\text{-}3}$, Gabrg1,3 – $\gamma_{1,3}$, Gabrd – δ, Gabre – ε, Gabrp – π, Gabrq – θ, Gabrr1-3 – $\rho_{1\text{-}3}$.

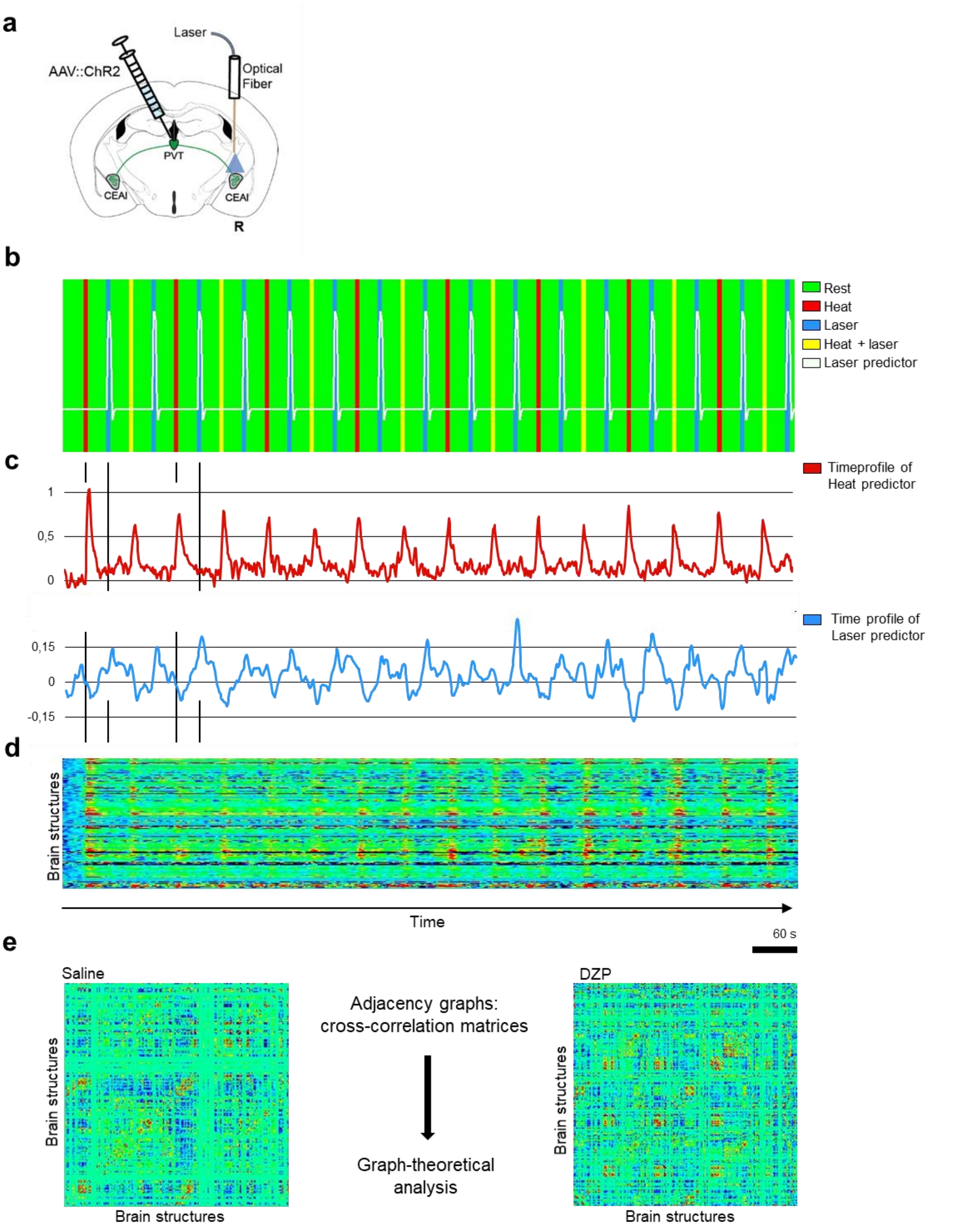

Supplementary Fig. S5 Optogenetic fMRI experiment setup and analysis workflow.
a AAV mediated expression of ChR2 in the PVT and optical fiber placement above the right CEAl.. **b** Temporal stimulus presentation sequence with the different stimuli, white line represents laser predictor. **c** Average time profiles as percentage BOLD signal change determined by the predictors heat and laser demonstrating the highly consistent coupling to the repetitive stimulation. Note the systematic temporal shift between heat and laser stimulation. **d** Representative temporal profiles for all brain structures analyzed (y axis) forming the basis for the following graph-theoretical analysis (see methods). **e** Resulting cross correlation (adjacency) matrices for saline (left) and DZP (right) subject to further analysis, see Supplementary Fig. S6.

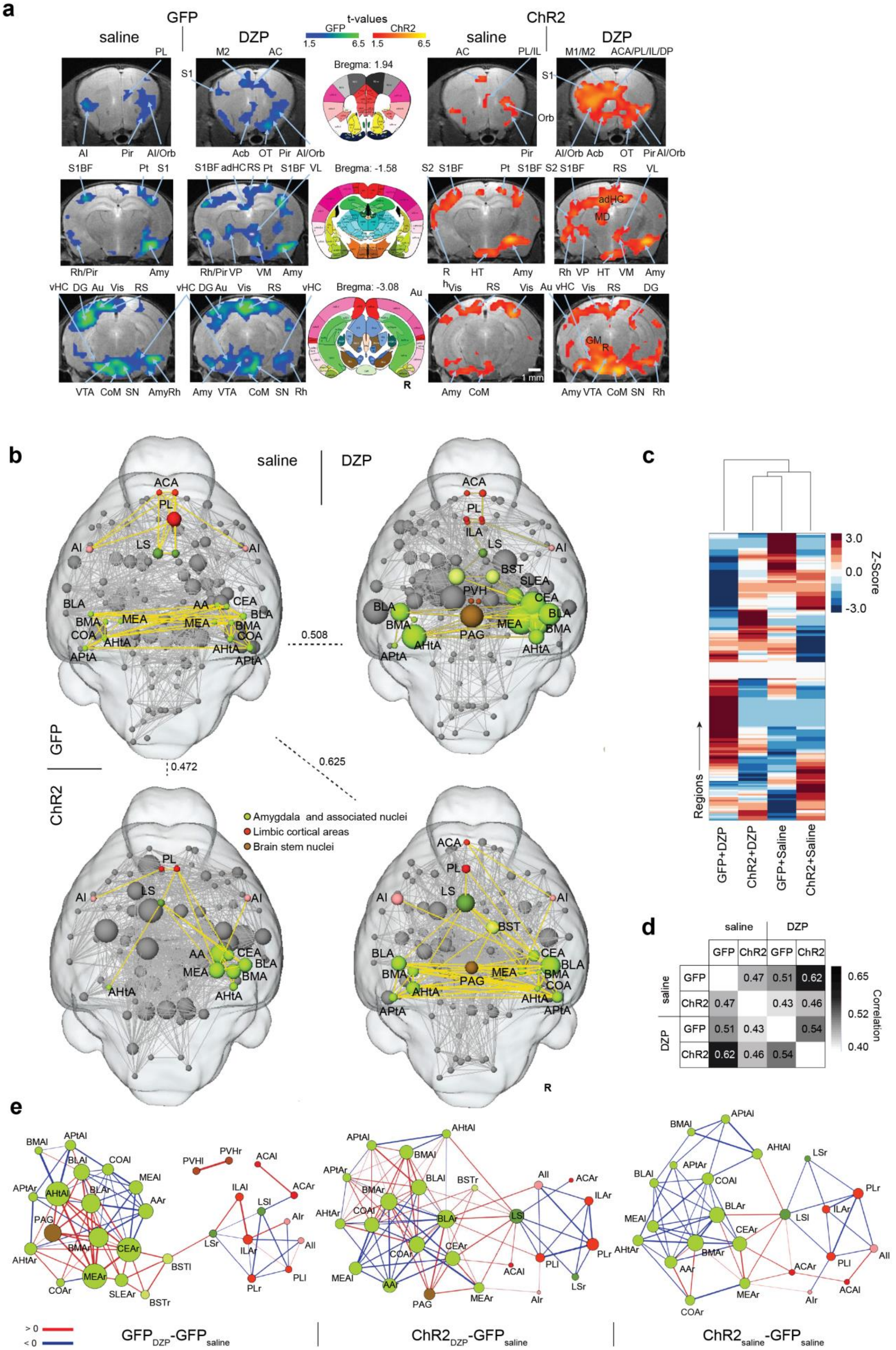
a
GFP
saline
DZP
ChR2
saline
DZP
t-values
GFP
ChR2
1.5
6.5
1.5
6.5
Bregma: 1.94
Bregma: -1.58
Bregma: -3.08
R
1 mm
b
saline
DZP
GFP
ChR2
0.508
0.472
0.625
Amygdala and associated nuclei
Limbic cortical areas
Brain stem nuclei
R
c
3.0
0.0
-3.0
Z-Score
Regions
GFP+DZP
ChR2+DZP
GFP+Saline
ChR2+Saline
d
saline
DZP
GFP
ChR2
GFP
ChR2
0.47
0.51
0.62
0.43
0.46
0.54
0.65
0.52
0.40
Correlation
e
> 0
< 0
GFP$_{DZP}$-GFP$_{saline}$
ChR2$_{DZP}$-GFP$_{saline}$
ChR2$_{saline}$-GFP$_{saline}$

Supplementary Fig. S6 Optogenetic activation of PVT-CEAl modulates aversive brain state globally and is modulated by DZP. **a** BOLD activation patterns for the GFP and ChR2 with saline and DZP treatments at 3 representative positions (Bregma 1.94, -1.58 and – 3.08) as a result of the initial GLM group analysis. **b** Brain-wide BOLD fMRI functional networks recruited by optogenetic activation of PVT-CEAl projections and systemic DZP application together with corresponding GFP controls. Animals virally expressing GFP (n = 4) or ChR2 (n = 3) in the PVT were laser-stimulated in the CEAl. The limbic functional subnetwork is colorized. Node size codes for sum of all edges, i.e. significant connections of that node. A 3D surface of an average mouse brain was rendered transparently. Dashed lines depict the overall network similarities of **d**. **c** Cluster analysis of brain-wide functional networks in **c** by column-normalized node strength. **d** Similarity assessment of the networks in **c** across the different experimental groups. Values are Pearson correlation coefficients of the respective connectivity matrices. **e** Kamadai-Kawai plots of differential networks, i.e. subtracting the GFP saline as control, representing DZP effects (left), effects of PVT-CEl stimulation (right) and the interaction of the two (middle). Here, the node-size represents the sum of the absolute differences of all edges of that node. The thickness of an edge codes its weight or the absolute difference in connectivity strength. Red edges indicate an increase of connectivity strength compared to GFP saline and blue edges a decrease. Note that DZP treatment rearranges functional coupling of the amygdala network and increases ILA, amygdala and PAG interaction (left). This is partially reverted by PVT-CEAl optogenetic activation under DZP treatment (middle), which reduces intra-amygdala, ILA, and PAG dominance (right). Increased interaction between amygdala and PAG may arise through DZP suppression, while PVT-CEAl activation activates the CEAm inhibitory output to the brainstem. Color code in **b, e** represents anatomical groups. Size represents node strength or correlation **b** and absolute differences of functional connectivity **e**. **R** indicates right hemisphere. AA – anterior amygdala; ACA – anterior cingulate area; Acb – nucleus accumbens; adHC – anteriodorsal hippocampus; AHtA – amygdala-hippocampus transition area; AI – agranular insular cortex; Amy – amygdala; APtA – amygdala-piriform transition area; Au – auditory cortex; BLA – basolateral amygdala; BMA – basomedial amygdala; BST – bed nucleus of the stria terminalis; CEA – central amygdala, CEAl – central amygdala lateral subdivision; CEAm – central amygdala, medial subdivision; COA – cortical amygdala; CoM – mammillary bodies; DG – dentate gyrus; ILA – infralimbic area; LS – lateral and medial septum; M2 – secondary motor cortex; MEA – medial amygdala; Orb – orbitofrontal cortex; OT – olfactory tubercle; PAG – periaqueductal gray; Pir – piriform cortex; PL – prelimbic area; Pt – parietal association cortex;PVH – paraventricular hypothalamus; Rh – entorhinal cortex; RS – retrosplenial cortex; S1 – primary sensory cortex; S1BF – primary sensory cortex barrel field; S2 – secondary somatosensory cortex; SLEA – sublenticular extended amygdala; SN – substantia nigra; vHC – ventral hippocampus; Vis – visual cortex; VTA – ventral tegmental area.

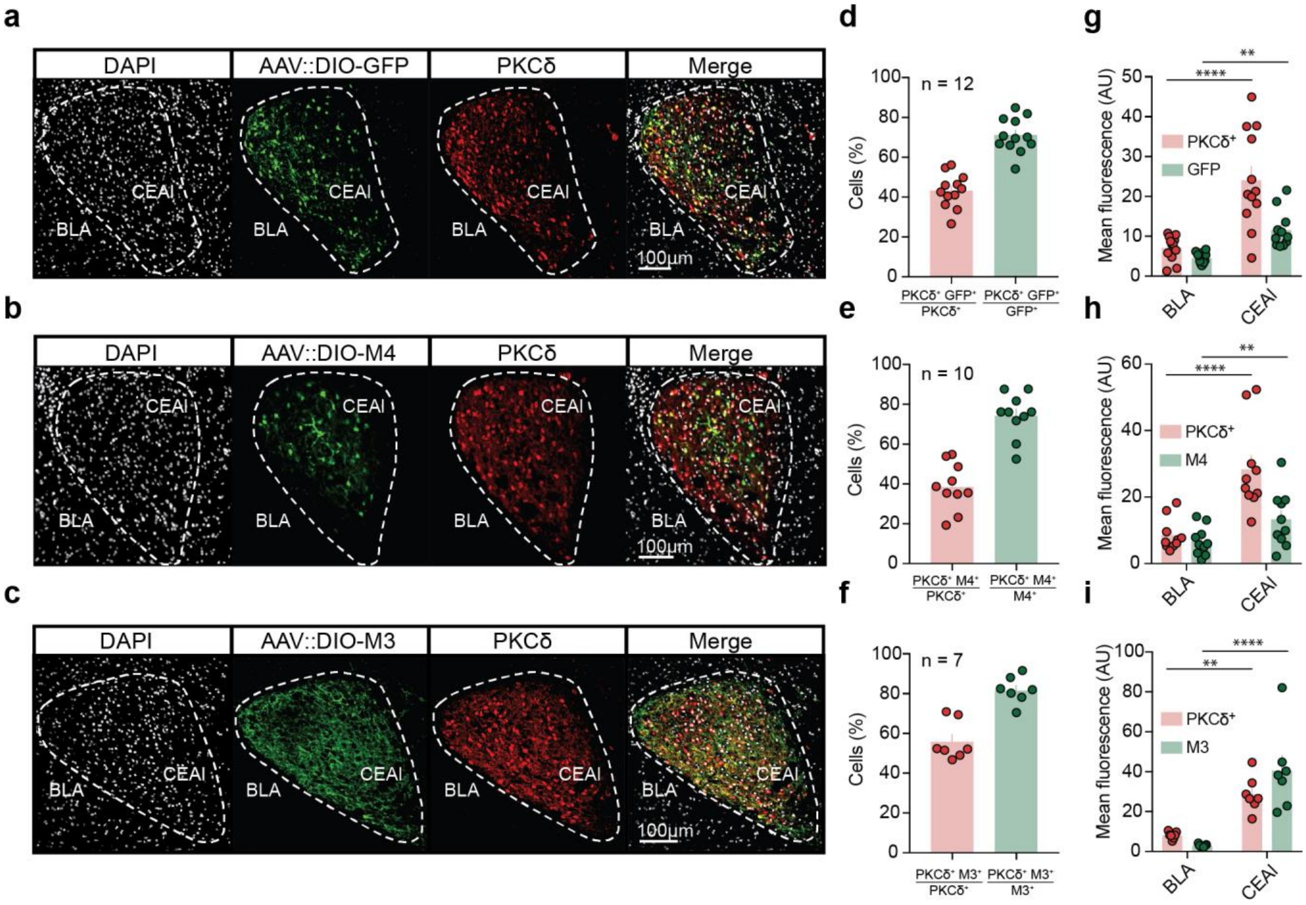

Supplementary Fig. S7 Virus expression in CEAl-DREADD cohorts.
a-c Confocal scans showing typical expression of AAV::DIO-GFP (**a**), AAV::DIO-M4 (**b**) and AAV::DIO-M3 (**c**) in CEAl. **d-i** Quantification of virus expression and spread in a subset of all mice used in this study (cf. Table S3). **d-f** Quantification of infected PKCδ$^+$ cells as a percentage of either all PKCδ$^+$ cells or all infected cells of AAV::DIO-GFP (**d**), AAV::DIO-M4 (**e**) and AAV::DIO-M3 (**f**) cohorts. **g-i** Mean fluorescence of PKCδ$^+$ and virus markers in BLA and CEAl. **g** AAV::DIO-GFP cohort (RM two-way ANOVA $F_{interaction}$ (1, 21) = 8.433, P = 0.0085; F_{region} (1, 21) = 50.84, P < 0.0001; F_{marker} (1, 21) = 11.11, P = 0.0032; Two-stage linear step-up procedure of Benjamini, Krieger and Yekutieli). **h** AAV::DIO-M4 cohort (RM two-way ANOVA $F_{interaction}$ (1, 18) = 17.93, P = 0.0005; F_{region} (1, 18) = 72.01, P < 0.0001; F_{marker} (1, 18) = 5.993, P = 0.0248; Two-stage linear step-up procedure of Benjamini, Krieger and Yekutieli). **i** AAV::DIO-M3 cohort (RM two-way ANOVA $F_{interaction}$ (1, 12) = 4.359, P = 0.0588; F_{region} (1, 12) = 51.3, P < 0.0001; F_{marker} (1, 12) = 0.559, P = 0.4691; Two-stage linear step-up procedure of Benjamini, Krieger and Yekutieli). Note that in all cohorts virus marker fluorescence is higher in CEAl than in BLA, and that virus fluorescence in the BLA is never above PKCδ-background, indicating that the virus did not spread outside the CEAl. Bars are means ± s.e.m. Significance levels between groups at * Q < 0.05, ** Q < 0.01, *** Q < 0.001, **** Q < 0.0001.

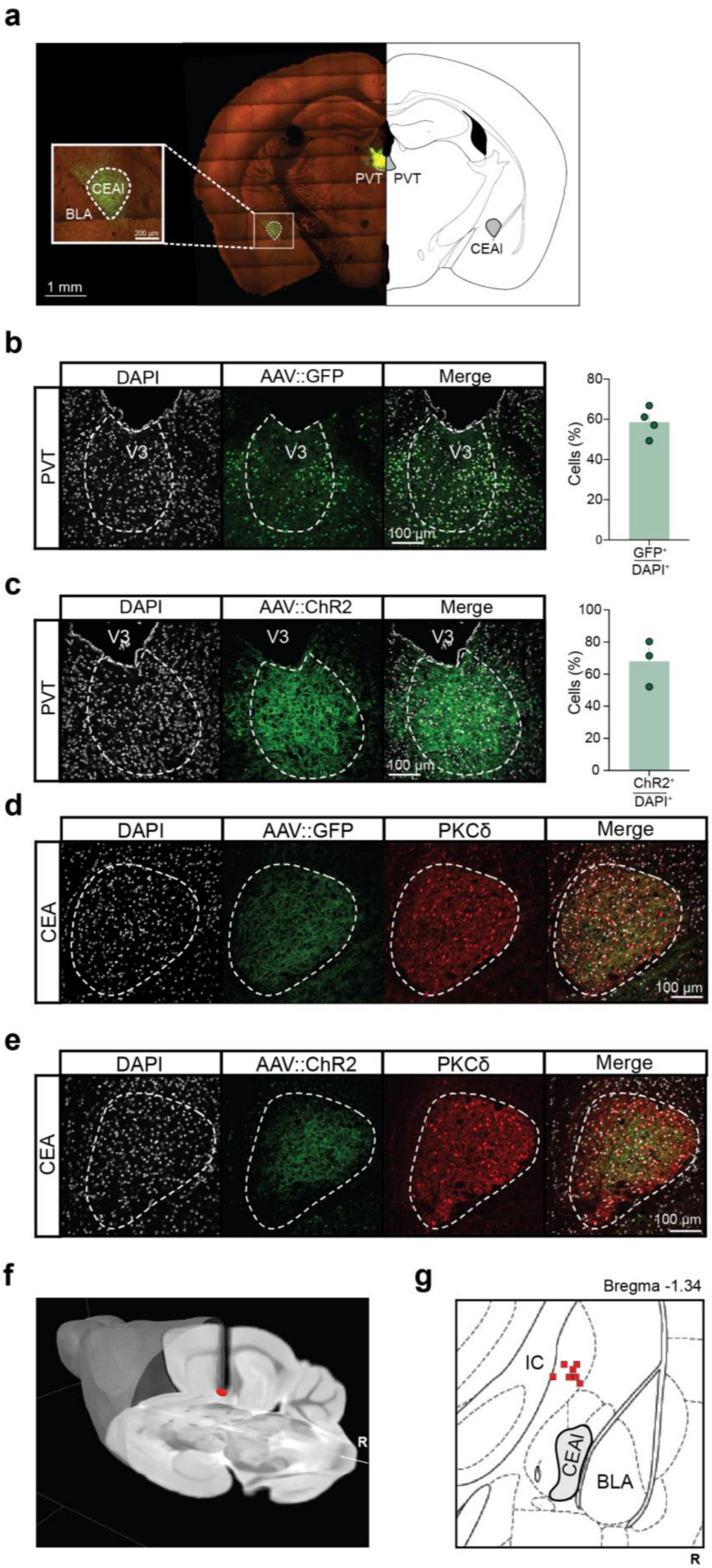
a
CEAl
BLA
200 µm
PVT
PVT
CEAl
1 mm
b
DAPI
AAV::GFP
Merge
PVT
V3
V3
V3
100 µm
Cells (%)
80
60
40
20
0
GFP⁺/DAPI⁺
c
DAPI
AAV::ChR2
Merge
PVT
V3
V3
V3
100 µm
Cells (%)
100
80
60
40
20
0
ChR2⁺/DAPI⁺
d
DAPI
AAV::GFP
PKCδ
Merge
CEA
100 µm
e
DAPI
AAV::ChR2
PKCδ
Merge
CEA
100 µm
f
R
g
Bregma -1.34
IC
CEAl
BLA
R

Supplementary Fig. S8 Virus expression and optical fiber placement in fMRI cohorts.
a AAV::EGFP injections into the PVT of C57BL/6 mice. Data taken from Allen Mouse Brain Connectivity Atlas. Experiment number: 120875111 (http://connectivity.brain-map.org). **b,c** Confocal scans showing typical expression (left), as well as infection rate (right) of AAV::GFP (**b**) and AAV::ChR2 (**c**). **d,e** Confocal scans showing typical PVT-CEAl projections in AAV::GFP (**d**) and AAV::ChR2 (**e**) injected animals. Note that terminal fields of virally infected PVT neurons within the temporal lobe are highly specific for the CEAl (**a,d,e**). **f,g** Optical fiber placement. **f** 3D reconstruction of average fiber placement in the experimental animals. Red area indicates the 3D standard deviation of fiber positions. Of note, the representation of the optical fiber in MR images is considerably overestimated due to susceptibility artefacts of the fiber itself. The fiber positions were determined from the anatomical reference scans for each animal. **g** Location of the fiber tip in each animal. **R** indicates right hemisphere.

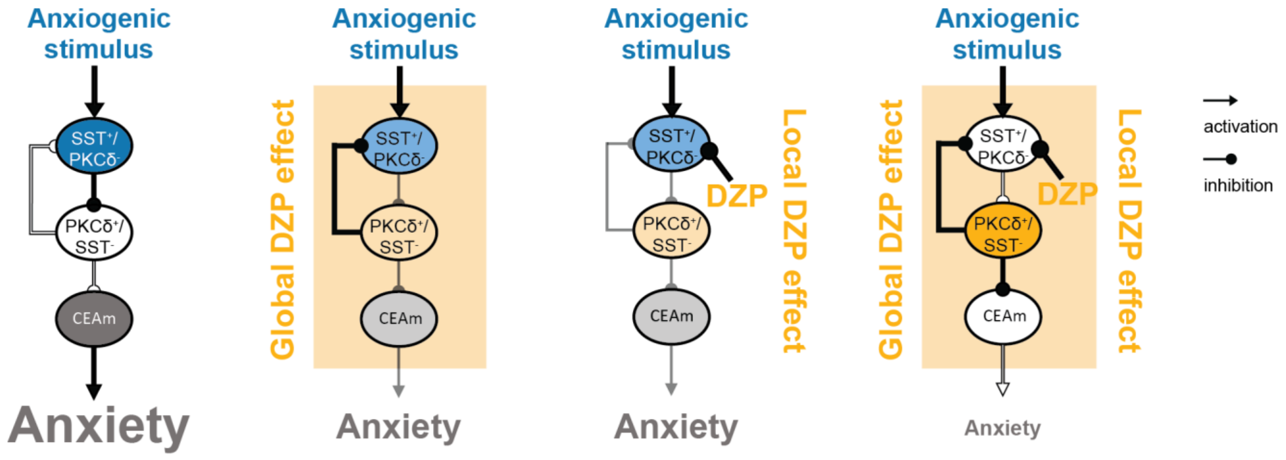

Supplementary Fig. S9 A circuit framework for the BZD anxiolytic effect.
Our proposed model of CEA circuitry and BZD anxiolytic effect, representing the major neuronal types in CEAl. We find that DZP modulates CEA signaling both by global (center-left) and local (center-right) mechanisms, which synergize (right) to promote an anxiolytic state in CEA circuitry. Inhibitory interactions within CEA may underlie these dynamic changes.

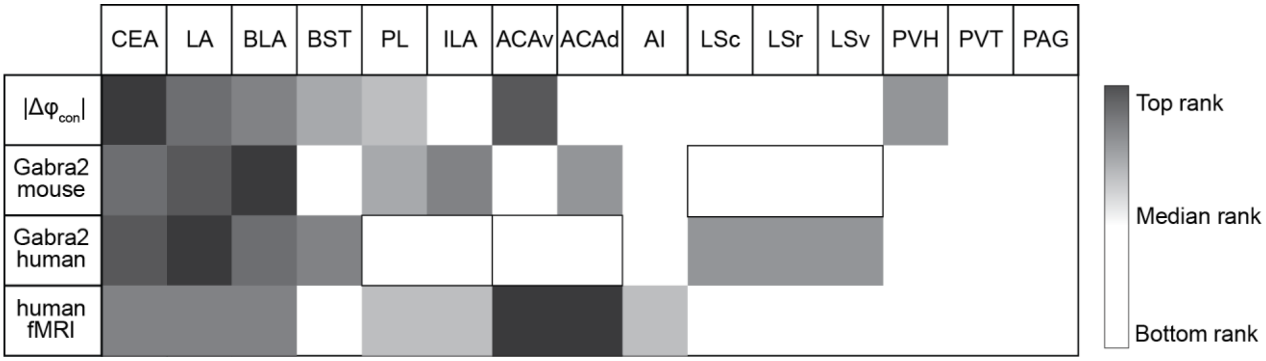

Supplementary Figure S10 Translational evaluation of the BZD anxiolytic mechanism.
Evaluation of amygdala inhibitory gating for the BZD anxiolytic effect in mice and humans. Note the strong amygdala interaction with DZP ($|\Delta\varphi_{con}|$) in mice, high expression of the ($GABA_A$-α_2) subunits in mice (cf. Fig. 2k) and humans (data from Allen Mouse , http://mouse.brain-map.org and Allen Human Brain Databases, http://human.brain-map.org), and evidence from the literature (see methods) for the amygdala mediating BZD anxiolytic effect from human fMRI (gray). Regions were ranked based on the absolute values in each category. Grey value intensity indicates rank above the median for functional connectivity, gene expression data and literature evidence (see methods). Boxes indicate where regions were pooled due to lower anatomical resolution in databases or literature. Human $GABA_A$-α_2 expression includes the medial septal nuclei. ACAd – anterior cingulate area, dorsal part; ACAv – anterior cingulate area, ventral part; AI – agranular insular area; BLA – basolateral amygdalar nucleus; BST – bed nuclei of the stria terminalis; CEA – central amygdalar nucleus; ILA – infralimbic area; LA – lateral amygdalar nucleus; LSc – lateral septal nucleus, caudodorsal part; LSr – lateral septal nucleus, rostroventral part; LSv – lateral septal nucleus, ventral part; PAG – periaqueductal gray; PL – prelimbic area; PVH – paraventricular hypothalamic nucleus; PVT – paraventricular nucleus of the thalamus. Gabra2 - $GABA_A$ receptor subunit α_2.

**Virus**	**Short name**	**Manufacturer**	**Titer (GC/ml)**
$AAV_{2/5}$-EF1α-DIO-GFP-WPRE-hGh	AAV::DIO-GFP	IMP	1×10^{11}
$AAV_{2/5}$-hSyn-DIO-hM3D-mCherry-WPRE-hGh	AAV::DIO-M3	University of Pennsylvania	2×10^{12}
$AAV_{2/5}$-hSyn-DIO-hM4D-mCherry-WPRE-hGh	AAV::DIO-M4	IMP	4×10^{11}
$AAV_{2/5}$-hSyn-eGFP-WPRE-hGh	AAV::GFP	University of Pennsylvania	1×10^{13}
$AAV_{2/5}$-hSyn-hChR2(H134R)-eYFP-WPRE-hGh	AAV::ChR2	University of Pennsylvania	2×10^{13}
AAV_{1}-Syn-Flex-GCaMP6f-WPRE-SV40	AAV::Flex-GCaMP6	University of Pennsylvania	1×10^{13}
AAV_{9}-hSyn-GCaMP6m-WPRE	AAV::GCaMP6	Boehringer Ingelheim	1×10^{13}

Supplementary Table S1 Viral constructs and abbreviations.
Full and short names of viruses used with manufacturer and obtained titer.

Reference	Drug	Experiment	Regions upregulated (BOLD)	Regions downregulated (BOLD)	Region scored for Fig. S10
Paulus et al. 2005 (17)	lorazepam	2 doses of lorazepam during fMRI session	-	amygdala, insula	**CEA, LA, BLA AI**
Schunck et al. 2010 (18)	lorazepam	anxiety symptom provocation challenge	-	Superior frontal gyrus, anterior/inferior frontal gyrus, cingulate gyrus	**PL, ILA, ACAv, ACAd**
Gospic et al. 2011 (19)	oxazepam	Ultimatum Game (Rejection of unfair proposals)	amygdala (in control group)	amygdala (in lorazepam group)	**CEA, LA, BLA**
Mathis, Erb, and Namer 2011 (20)	lorazepam	CCK-4 induced anxiety	cingulate gyrus (in control group)	cingulate gyrus (in lorazepam group)	**ACAv, ACAd**
Del-ben et al. 2012 (21)	diazepam	presentation of aversive faces	right anterior cingulate cortex (fearful faces)<br><br>posterior left insula (angry faces)	right amygdala, right orbitofrontal cortex (fearful faces)<br><br>bilateral anterior, cingulate cortex (angry faces)	**CEA, LA BLA PL, ILA ACAv, ACAd**
Leicht et al. 2013 (22)	alprazolam	CCK-4 induced anxiety	-	rostral anterior cingulate cortex (blocks increase by CCK-4)	**ACAv, ACAd**
Pflanz et al. 2014 (23)	diazepam	7-day diazepam administration	increased functional connectivity in medial visual network and medial/inferior temporal network	-	-
Brown et al. 2015 (24)	alprazolam	effect on patients with generalized anxiety disorder	-	amygdala, anterior insula	**CEA, LA, BLA AI**
Walter et al. 2016 (25)	diazepam	working memory task	-	cingulate cortex (inversely correlated with diazepam blood-concentration)	**ACAv, ACAd**

Supplementary Table S2 fMRI literature search results.
Results from our literature search for studies on BZDs using BOLD fMRI in humans with a brief summary of each study's findings and our selected regions scored for Fig. S10. ACAd – anterior cingulate area, dorsal part; ACAv – anterior cingulate area, ventral part; AI – agranular insular area; BLA – basolateral amygdalar nucleus;; CEA – central amygdalar nucleus; ILA – infralimbic area; LA – lateral amygdalar nucleus; PL – prelimbic area;

Virus	Target region	Nr. of mice injected	Virus expression	No virus expression
AAV::DIO-GFP	CEAl	19	18	1
AAV::DIO-M4	CEAl	18	15	3
AAV::DIO-M3	CEAl	8	7	1
AAV::GFP	PVT	4	4	0
AAV::ChR2	PVT	3	3	0

Supplementary Table S3 Classification of virus expression.
Post-mortem histological classification of all virus-injected mice used in this study. Mice that did not show virus expression were excluded from all data analyses.

Region		ACAd	ACAv	AI	BLA	BST	CEA	ILA	LA	LSc	LSr	LSv	PAG	PL	PVH	PVT
**home cage**		3	3	3	4	2	5	2	4	3	2	3	4	2	5	5
**injection only**	**saline**	7	7	7	5	5	6	5	5	6	6	5	4	5	4	6
	**DZP**	5	5	6	5	4	5	5	5	5	5	5	6	5	5	7
**EPM**	**saline**	6	5	8	7	4	8	5	7	6	6	6	8	4	4	8
	**DZP**	8	8	6	7	7	7	7	7	8	8	8	6	5	9	9
**shock**	**saline**	5	5	6	7	5	7	3	7	6	6	6	5	3	7	7
	**DZP**	4	4	5	5	4	5	5	5	5	5	5	6	5	5	5
**shock & EPM**	**saline**	6	6	8	8	5	9	5	8	5	5	5	9	6	8	9
	**DZP**	5	5	8	7	3	7	3	7	3	3	3	5	5	5	7

Supplementary Table S4 Sample number of the c-Fos screen.
Sample number for each region and condition in the c-Fos screen. A sample number of n indicates samples from n different animals, each from at least 2 histological slices. Please note that the 'home cage' group was not used for any analysis, but only for illustration in Fig. 1b. ACAd – anterior cingulate area, dorsal part; ACAv – anterior cingulate area, ventral part; AI – agranular insular area; BLA – basolateral amygdalar nucleus; BST – bed nuclei of the stria terminalis; CEA – central amygdalar nucleus; ILA – infralimbic area; LA – lateral amygdalar nucleus; LSc – lateral septal nucleus, caudodorsal part; LSr – lateral septal nucleus, rostroventral part; LSv – lateral septal nucleus, ventral part; PAG – periaqueductal gray; PL – prelimbic area; PVH – paraventricular hypothalamic nucleus; PVT – paraventricular nucleus of the thalamus

Supplementary References

1. Haubensak W, Kunwar PS, Cai H, Ciocchi S, Wall NR, Ponnusamy R, et al. Genetic dissection of an amygdala microcircuit that gates conditioned fear. Nature. 2010 Nov 11;468(7321):270-6. PubMed PMID: 21068836. Epub 2010/11/12. eng.
2. Athos J, Storm DR. High precision stereotaxic surgery in mice. Current protocols in neuroscience / editorial board, Jacqueline N Crawley [et al]. 2001 May;Appendix 4:Appendix 4A. PubMed PMID: 18428449.
3. Gomez JL, Bonaventura J, Lesniak W, Mathews WB, Sysa-Shah P, Rodriguez LA, et al. Chemogenetics revealed: DREADD occupancy and activation via converted clozapine. Science. 2017 Aug 4;357(6350):503-7. PubMed PMID: 28774929. Epub 2017/08/05.
4. Fanselow MS. Conditioned and unconditional components of post-shock freezing. The Pavlovian journal of biological science. 1980 Oct-Dec;15(4):177-82. PubMed PMID: 7208128.
5. Franklin KBJ, Paxinos G. The mouse brain in stereotaxic coordinates. 3rd ed ed. Amsterdam: Boston : Elsevier/Academic Press; 2008.
6. Schindelin J, Arganda-Carreras I, Frise E, Kaynig V, Longair M, Pietzsch T, et al. Fiji: an open-source platform for biological-image analysis. Nature methods. 2012 Jun 28;9(7):676-82. PubMed PMID: 22743772. Pubmed Central PMCID: 3855844.
7. Panhelainen AE, Korpi ER. Evidence for a role of inhibition of orexinergic neurons in the anxiolytic and sedative effects of diazepam: A c-Fos study. Pharmacology, biochemistry, and behavior. 2012 Mar;101(1):115-24. PubMed PMID: 22210490.
8. Linden AM, Greene SJ, Bergeron M, Schoepp DD. Anxiolytic activity of the MGLU2/3 receptor agonist LY354740 on the elevated plus maze is associated with the suppression of stress-induced c-Fos in the hippocampus and increases in c-Fos induction in several other stress-sensitive brain regions. Neuropsychopharmacology. 2004 Mar;29(3):502-13. PubMed PMID: 14694349.
9. Herry C, Ciocchi S, Senn V, Demmou L, Muller C, Luthi A. Switching on and off fear by distinct neuronal circuits. Nature. 2008 Jul 31;454(7204):600-6. PubMed PMID: 18615015.
10. Ritov G, Boltyansky B, Richter-Levin G. A novel approach to PTSD modeling in rats reveals alternating patterns of limbic activity in different types of stress reaction. Molecular psychiatry. 2016 May;21(5):630-41. PubMed PMID: 26552592. Pubmed Central PMCID: 5414084.
11. Li H, Durbin R. Fast and accurate short read alignment with Burrows-Wheeler transform. Bioinformatics. 2009 Jul 15;25(14):1754-60. PubMed PMID: 19451168. Pubmed Central PMCID: 2705234.
12. Bray NL, Pimentel H, Melsted P, Pachter L. Near-optimal probabilistic RNA-seq quantification. Nature biotechnology. 2016 May;34(5):525-7. PubMed PMID: 27043002.
13. Pliota P, Bohm V, Grossl F, Griessner J, Valenti O, Kraitsy K, et al. Stress peptides sensitize fear circuitry to promote passive coping. Molecular psychiatry. 2018 Jun 14. PubMed PMID: 29904149. Epub 2018/06/16.
14. Lee JH, Durand R, Gradinaru V, Zhang F, Goshen I, Kim DS, et al. Global and local fMRI signals driven by neurons defined optogenetically by type and wiring. Nature. 2010 Jun 10;465(7299):788-92. PubMed PMID: 20473285. Pubmed Central PMCID: PMC3177305. Epub 2010/05/18.
15. Thompson SJ, Bushnell MC. Rodent functional and anatomical imaging of pain. Neuroscience letters. 2012 Jun 29;520(2):131-9. PubMed PMID: 22445887.
16. Schroeter A, Schlegel F, Seuwen A, Grandjean J, Rudin M. Specificity of stimulus-evoked fMRI responses in the mouse: the influence of systemic physiological changes associated with innocuous stimulation under four different anesthetics. NeuroImage. 2014 Jul 01;94:372-84. PubMed PMID: 24495809.
17. Paulus MP, Feinstein JS, Castillo G, Simmons AN, Stein MB. Dose-Dependent Decrease of Activation in Bilateral Amygdala and Insula by Lorazepam During Emotion Processing. 2005;62.
18. Schunck T, Mathis A, Erb G, Luthringer R. Effects of lorazepam on brain activity pattern during an anxiety symptom provocation challenge. 2010.
19. Gospic K, Mohlin E, Fransson P, Petrovic P, Johannesson M. Limbic Justice — Amygdala Involvement in Immediate Rejection in the Ultimatum Game. 2011;9:1-8.
20. Mathis A, Erb G, Namer IJ. One milligram of lorazepam does not decrease anxiety induced by CCK-4 in healthy volunteers : Investigation of neural correlates with BOLD MRI. 2011.
21. Del-ben CM, Ferreira CAQ, Sanchez TA, Alves-neto WC, Guapo VG, Araujo DBD, et al. Effects of diazepam on BOLD activation during the processing of aversive faces. 2012.
22. Leicht G, Mulert C, Eser D, Sämann PG, Ertl M, Laenger A, et al. Benzodiazepines counteract rostral anterior cingulate cortex activation induced by cholecystokinin-tetrapeptide in humans. Biol Psychiatry. 2013;73:337-44. PubMed PMID: 23059050.
23. Pflanz CP, Pringle A, Filippini N, Warren M, Gottwald J, Cowen PJ, et al. Effects of seven-day diazepam administration on resting-state functional connectivity in healthy volunteers : a randomized , double-blind study. 2014.
24. Brown GG, Ostrowitzki S, Stein B, Kienlin MV, Liu TT, Wierenga C, et al. Temporal profile of brain response to alprazolam in patients with generalized anxiety disorder. Psychiatry Research: Neuroimaging. 2015.
25. Walter SA, Forsgren M, Lundeng K, Simon R. Positive Allosteric Modulator of GABA Lowers BOLD Responses in the Cingulate Cortex. 2016:1-17.
